# Supplementary figures and images for: Characterization of the basic helix–loop–helix gene family and its tissue-differential expression in response to salt stress in poplar
Source: PeerJ. 2018 Mar 14;6:e4502. doi: 10.7717/peerj.4502 (PMC5857177; doi:10.7717/peerj.4502)

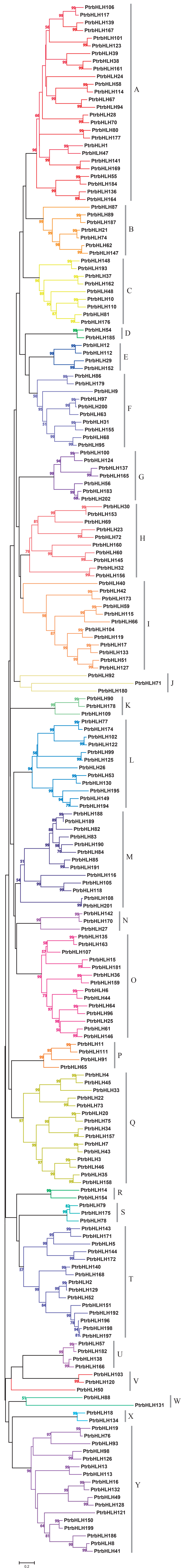

Supplement: Supplemental Information 4 [file peerj-06-4502-s004.pdf]

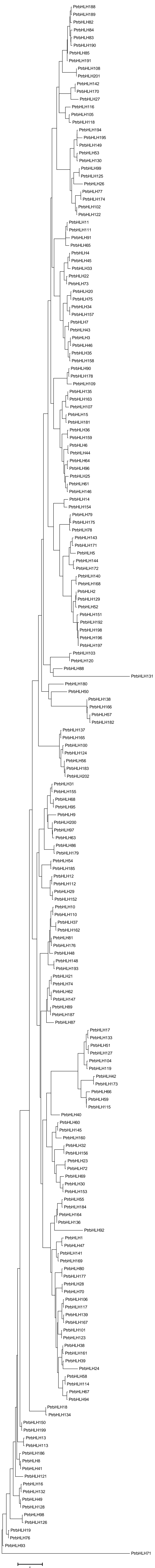

Supplement: Supplemental Information 5 [file peerj-06-4502-s005.pdf]

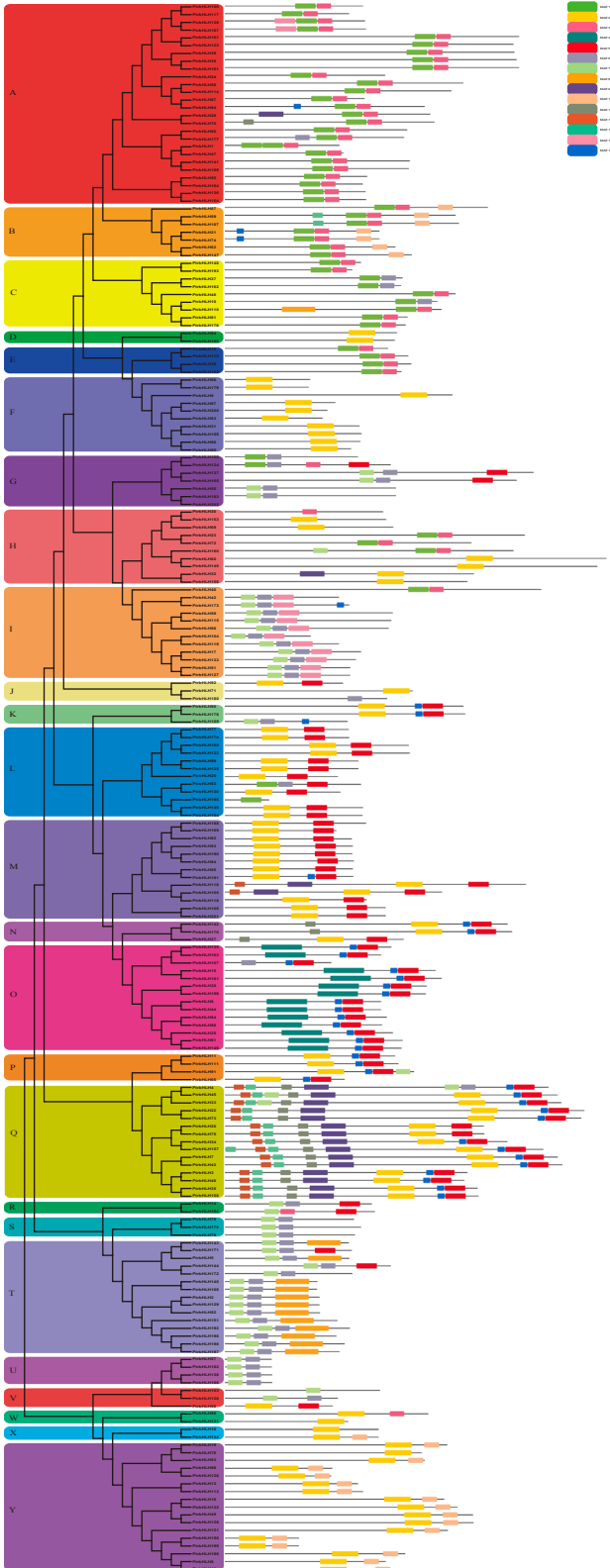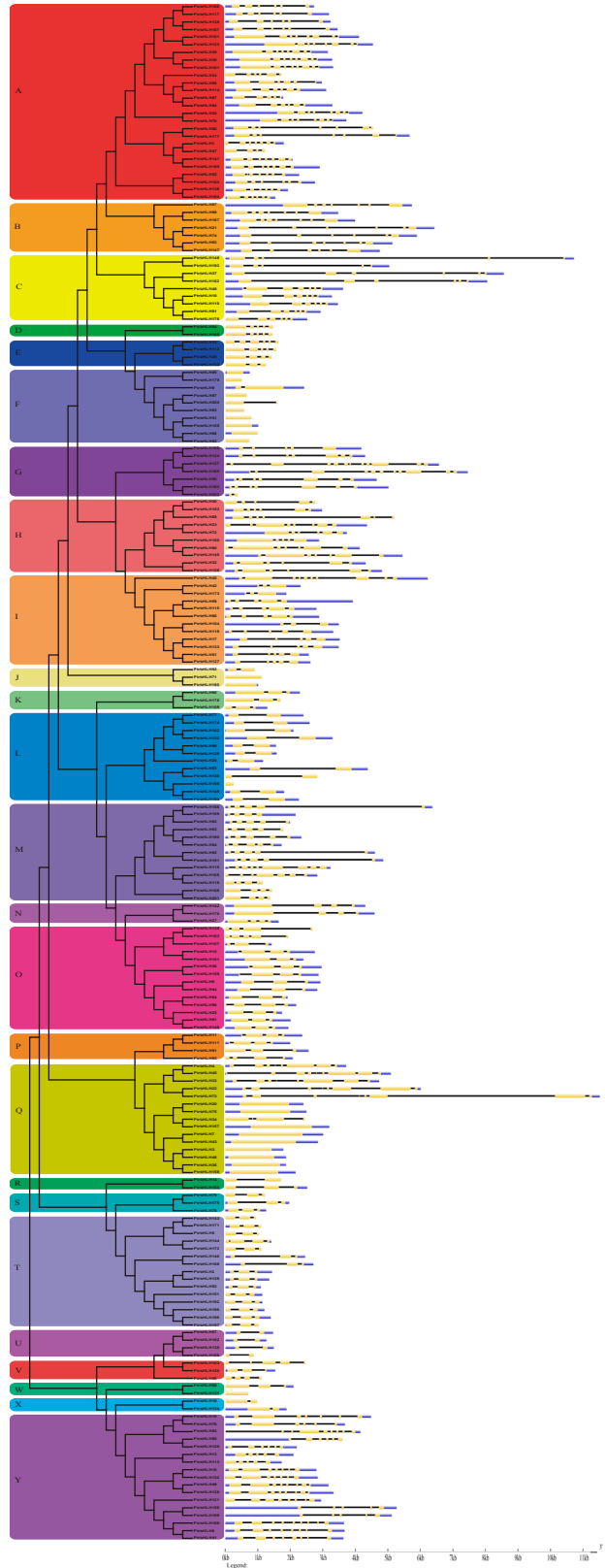

Supplement: Supplemental Information 6 — The groups are based on the phylogenetic tree. The colorful boxes represent different motifs. Yellow, black, and blue bars represent exons, introns, and 5’ UTR/3’ UTR, respectively. [file peerj-06-4502-s006.pdf]

**L(C)-R(C)**

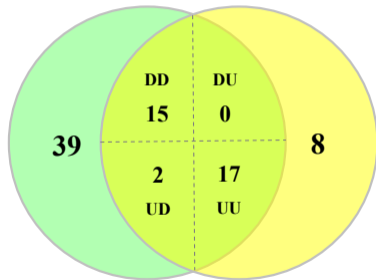

**L(C)-S(C)**

**R(C)-L(C)**

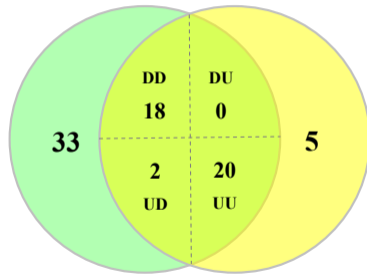

**R(C)-S(C)**

**S(C)-L(C)**

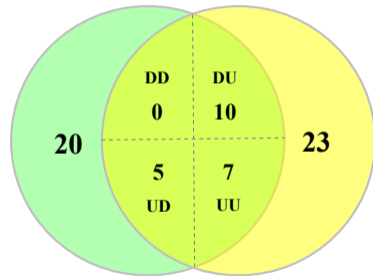

**S(C)-R(C)**

Supplement: Supplemental Information 7 — DD: down-regulated in the two comparisons. DU: down-regulated in the left comparison and up-regulated in the right comparison. UD: up-regulated in the left comparison and down-regulated in the right comparison. UU: up-regulated in the two comparisons. The variations of gene expressions occurred in leaves, roots and stems orderly. [file peerj-06-4502-s007.pdf]

log2 FoldChange

0

-10

-20

Leaf

Root

Stem

Regulation

down

up

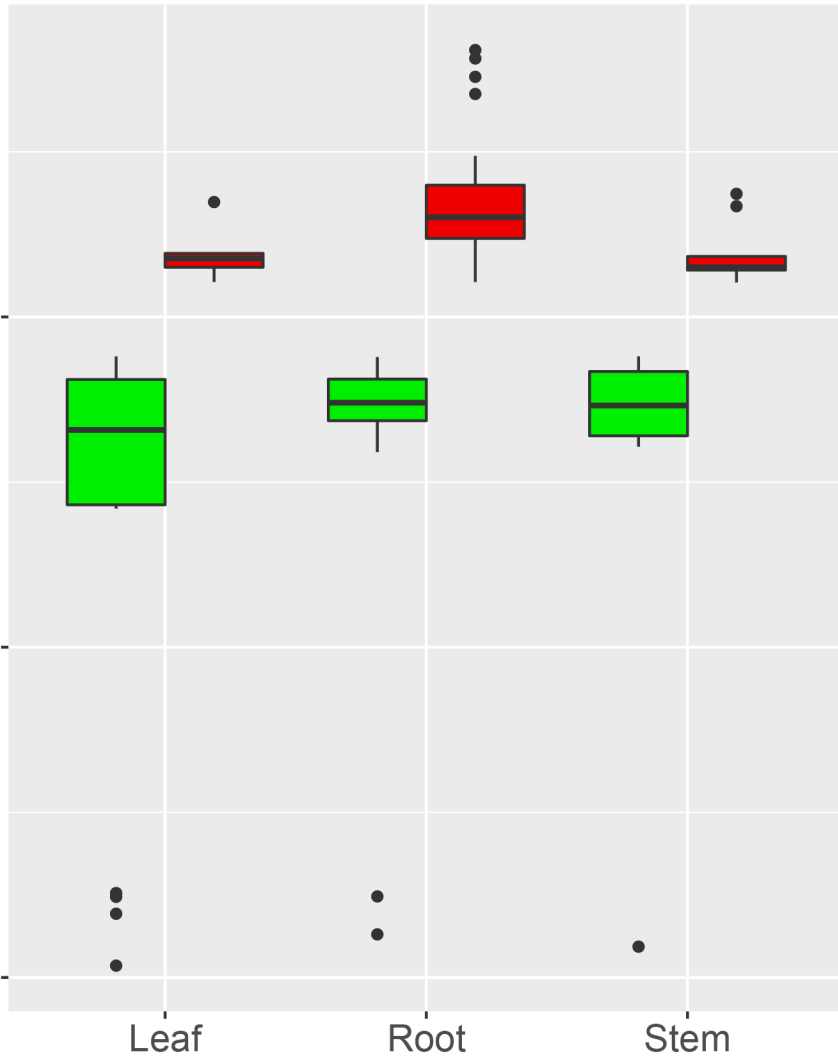

Supplement: Supplemental Information 8 — We computed log2 ratio of the FPKM values corresponding to the salt treatment and the control for each DEG. The boxplots display the median and variation of the log2 ratio, based on the DEGs, for each tissue. [file peerj-06-4502-s008.pdf]

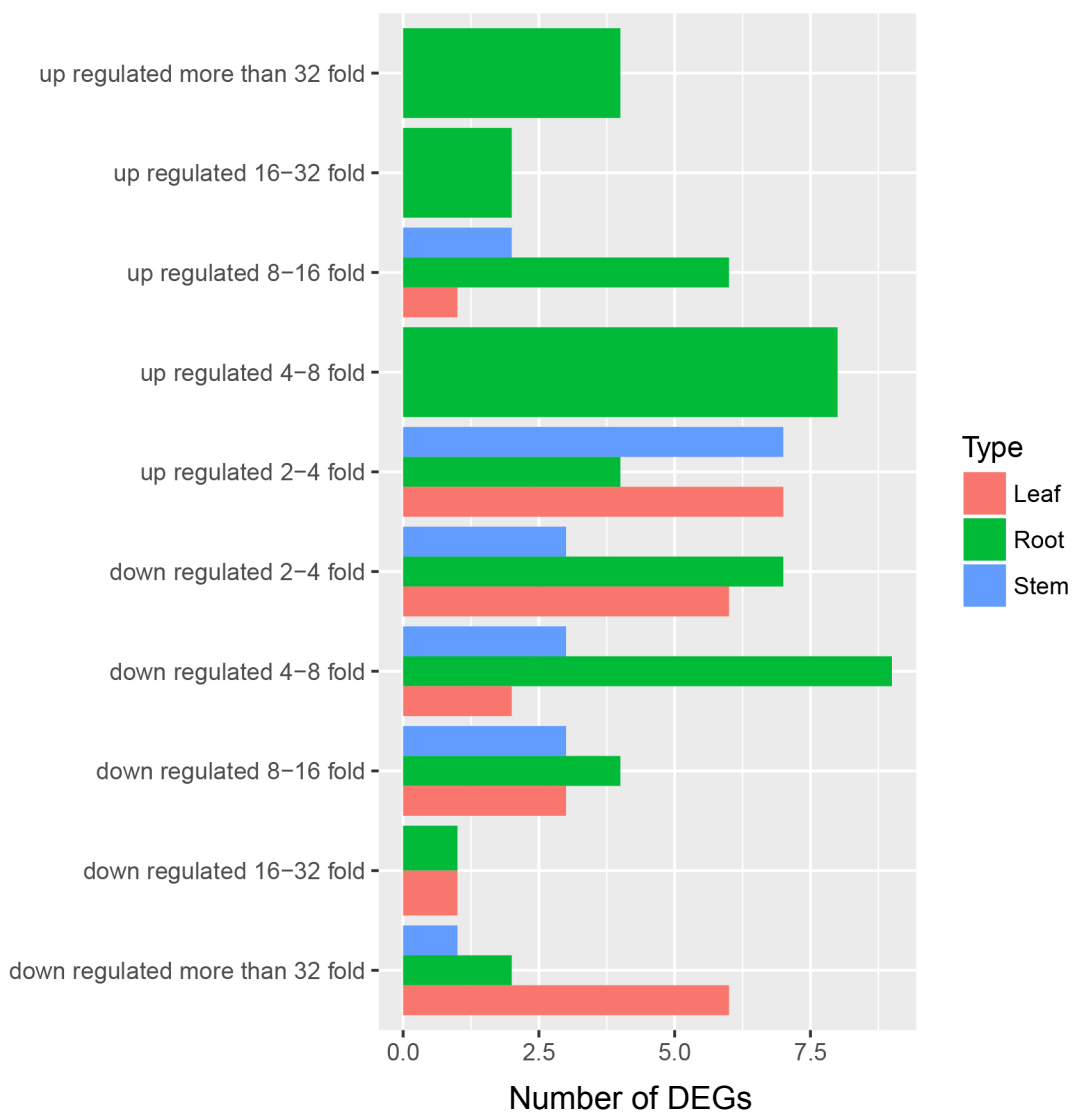

Supplement: Supplemental Information 9 — We grouped the URGs and DRGs into ten categories based on the relative gene expression. [file peerj-06-4502-s009.pdf]
