# Supplementary material for: Characterization of the basic helix–loop–helix gene family and its tissue-differential expression in response to salt stress in poplar
Source: PeerJ. 2018 Mar 14;6:e4502. doi: 10.7717/peerj.4502 (PMC5857177; doi:10.7717/peerj.4502)
Supplement: Supplemental Information 10 [file peerj-06-4502-s010.doc]

Primers sequence for quantitative real-time PCR

| Name | ID | 5’ primers | 3’ primers |
| --- | --- | --- | --- |
| ACT | JM986590 | ACCCTCCAATCCAGACACTG | TTGCTGACCGTATGAGCAAG |
| DEG1 | Potri.009G117300.1 | GTGGTTTAAGTGAGGTGGTGAC | GAGCCTTTCACTGTAGCCGCTG |
| DEG2 | Potri.018G083700.1 | GGTGGATTGGTGTGTGATGAAGAC | GACATCCATAACCGAAGCACTTG |
| DEG3 | Potri.016G051100.1 | CGTGGAGTTGCTTCATTCGGATTCG | GTCTCTAACGGCTCCCTCTTAAC |
| DEG4 | Potri.015G142700.1 | GCTCTCTTATCTGAATCTCGCTATG | CACTCTCGATTCCAGTTCACCCTGC |
| DEG5 | Potri.012G104900.1 | ACCGCTACTCCTACTACCTCCTTTG | TTCGCTCATCCACATCCCACATTAG |
| DEG6 | Potri.012G065000.1 | GGCAAGCCAAACTCAACCTCAATC | CTACTATTTCCTGTCTTCCCGAGC |
| DEG7 | Potri.012G055700.1 | GGCAATGGATGTGTCTGAAAGCAG | TCCCATCCTCATTGCTTTTCACTC |
| DEG8 | Potri.011G080000.1 | CAGAAGAGTGTGATGGGGAAGAGG | GCACCATAACTGGAGCAAGAATCAG |
| DEG9 | Potri.011G031000.1 | GTGATGAGAAACAGAGGCTAAAGAC | CCTGAGAGGTATCAACAACAGCAGG |
| DEG10 | Potri.010G137600.1 | CAGGAGAAGGTGCAAAAGTATGAGG | GCAGGAGCAGGCATGGGAAGAGC |
| DEG11 | Potri.014G111400.1 | CAGGTTCTTATGCGAGTATTGC | CTTGGCCTCTTTGTAGTGTAGC |
| DEG12 | Potri.010G136100.1 | GAGTGGAGAGGATGCTGAGCTTG | CCCTGTTTACCTTGTTTACCACTAG |
| DEG13 | Potri.009G064700.1 | CAGAAGGCGATGTTAGCGAGGCAAG | GTTCCCATCATTATCCCTCTGTC |
| DEG14 | Potri.008G165700.1 | GCTGAAAAGACTGAACTCCGTGAAG | CATGATAGGCTGCTGGATAGGTTGG |
| DEG15 | Potri.008G116000.1 | GGATGAGCTAACAGTGGATACAGC | GTGGTTGCTGTTGCTGTTGATGATC |
| DEG16 | Potri.008G070800.1 | CGTTCATCAGGGCTTCTGTTTGCTG | GCTTTTAACCCTTCCACCTACACTG |
| DEG17 | Potri.005G230800.1 | GCAAATCTCCATCACCAGCATC | CTATTGAGAGAAGAGATTGGAC |
| DEG18 | Potri.005G207200.1 | GCATCAGCATCAGCGACAACTCAAC | GCTTCACCAAATCCTCCTACACCAG |
| DEG19 | Potri.005G158100.1 | GTTGAAGATGATGGGATTGATATGC | CATCAAACCAATCTCGCCTATCATG |
| DEG20 | Potri.004G168100.1 | CCATTGCATTCATCCATACAGCC | GGCTGTGAAAGAGAAGAAACATG |
| DEG21 | Potri.005G208600.1 | GAGCCATCAGCCAATGCCGAAC | ATCATCAGGAGAACCAAGCCTG |
| DEG22 | Potri.010G186700.1 | CACTATTCTCCTTGCTGGCCTG | GAAAGTTGGCAGAGGCAGAAGC |
| DEG23 | Potri.004G156000.1 | CTCATCCTGCTTCACTCAAATCCAG | CTGAAAATGGCTGAACTGGGAAGGT |
| DEG24 | Potri.002G248500.1 | GGCTAAACAATGCTGAAATGGC | CTGGTGGGTTTGATGCTGATTG |
| DEG25 | Potri.002G172100.1 | TATGGTTGCTGCTGTTTTAGGCTC | CAACCAAGAACCCAATCACCAGAC |
| DEG26 | Potri.002G143300.1 | GATGGCTGGTATGGGAATGGGAATG | GCATGAAAGCAGTGGGGTGAAGAAC |
| DEG27 | Potri.002G114700.1 | GCAAACAATGCTCAACATCAGGAAG | CTCCTTCAGTATTCTTGTTTGGATC |
| DEG28 | Potri.002G055400.1 | TCAGAAGAACCCCATAGAGCCAGAC | GTCATAGCCCCTTACTGTTGGTG |
| DEG29 | Potri.002G054100.1 | TGCCACCTCATCAGACCATCCTTAC | ATCAACTCGCTCGGCTCAACTACTG |
| DEG30 | Potri.001G410600.1 | GCACCAATTATCAGCAAGAAACTGC | GGAATCTCCAGTGACCCCTGTGAAC |
