# Supplementary material for: Characterization of the basic helix–loop–helix gene family and its tissue-differential expression in response to salt stress in poplar
Source: PeerJ. 2018 Mar 14;6:e4502. doi: 10.7717/peerj.4502 (PMC5857177; doi:10.7717/peerj.4502)
Supplement: Supplemental Information 11 [file peerj-06-4502-s011.doc]

Physicochemical properties of bHLH proteins

| Name | Gene ID | Number of amino acids | Molecular weight | Theoretical pI | Aliphatic index | Grand average of hydropathicity (GRAVY) |
| --- | --- | --- | --- | --- | --- | --- |
| PtrbHLH1 | Potri.001G062900.1 | 222 | 25053.65 | 9.38 | 80.72 | -0.657 |
| PtrbHLH2 | Potri.001G063000.1 | 183 | 20500.85 | 5.84 | 76.17 | -0.659 |
| PtrbHLH3 | Potri.001G083500.1 | 471 | 52730.3 | 7.17 | 75.14 | -0.521 |
| PtrbHLH4 | Potri.001G103600.1 | 629 | 69928.42 | 6.23 | 92.03 | -0.406 |
| PtrbHLH5 | Potri.001G113400.1 | 241 | 27768.71 | 8.96 | 88.92 | -0.648 |
| PtrbHLH6 | Potri.001G141100.1 | 303 | 34246.27 | 8.22 | 90.79 | -0.32 |
| PtrbHLH7 | Potri.001G142200.1 | 647 | 71111.94 | 5.34 | 69.77 | -0.615 |
| PtrbHLH8 | Potri.001G185900.1 | 312 | 33845.38 | 5.22 | 64.39 | -0.564 |
| PtrbHLH9 | Potri.001G191800.1 | 442 | 48475.76 | 5.86 | 64.43 | -0.652 |
| PtrbHLH10 | Potri.001G270000.1 | 413 | 45737.75 | 9.15 | 56.22 | -0.826 |
| PtrbHLH11 | Potri.001G287200.1 | 330 | 36953.29 | 8.47 | 81.21 | -0.344 |
| PtrbHLH12 | Potri.001G294300.1 | 317 | 34568.25 | 5.37 | 64.64 | -0.776 |
| PtrbHLH13 | Potri.001G299300.1 | 258 | 27968.17 | 5.65 | 63.45 | -0.473 |
| PtrbHLH14 | Potri.001G305100.1 | 285 | 31949.87 | 5.95 | 71.16 | -0.567 |
| PtrbHLH15 | Potri.001G314400.1 | 409 | 46164.83 | 4.98 | 79.17 | -0.579 |
| PtrbHLH16 | Potri.001G410600.1 | 426 | 46886.56 | 6.26 | 53.8 | -0.676 |
| PtrbHLH17 | Potri.001G416600.1 | 264 | 29368.29 | 7.12 | 69.96 | -0.65 |
| PtrbHLH18 | Potri.001G461000.1 | 298 | 33413.57 | 4.77 | 75.27 | -0.434 |
| PtrbHLH19 | Potri.002G032400.1 | 432 | 47018.28 | 6.38 | 64.63 | -0.588 |
| PtrbHLH20 | Potri.002G042000.1 | 503 | 54943.08 | 6.59 | 78.07 | -0.39 |
| PtrbHLH21 | Potri.002G045400.1 | 300 | 31832.11 | 5.74 | 79.1 | -0.286 |
| PtrbHLH22 | Potri.002G054100.1 | 699 | 78304.6 | 4.76 | 77.84 | -0.569 |
| PtrbHLH23 | Potri.002G055400.1 | 583 | 64164.79 | 6.13 | 60.69 | -0.657 |
| PtrbHLH24 | Potri.002G100100.1 | 311 | 35451.23 | 9.89 | 89.61 | -0.419 |
| PtrbHLH25 | Potri.002G101900.1 | 326 | 36715.38 | 5.6 | 78.37 | -0.52 |
| PtrbHLH26 | Potri.002G105300.1 | 219 | 24038.25 | 9.21 | 72.1 | -0.588 |
| PtrbHLH27 | Potri.002G108400.1 | 347 | 39445.61 | 4.71 | 72.22 | -0.506 |
| PtrbHLH28 | Potri.002G114700.1 | 399 | 43473.31 | 5.57 | 66.94 | -0.665 |
| PtrbHLH29 | Potri.002G119200.1 | 362 | 40304.67 | 4.63 | 65 | -0.669 |
| PtrbHLH30 | Potri.002G124400.1 | 307 | 32466.07 | 4.88 | 71.82 | -0.311 |
| PtrbHLH31 | Potri.002G125000.1 | 261 | 29428.13 | 8.78 | 59.12 | -0.742 |
| PtrbHLH32 | Potri.002G143300.1 | 484 | 52715.58 | 8.8 | 54.83 | -0.592 |
| PtrbHLH33 | Potri.002G159400.1 | 654 | 73546.02 | 5.26 | 81.82 | -0.502 |
| PtrbHLH34 | Potri.002G172100.1 | 549 | 61068.59 | 6.82 | 73.52 | -0.527 |
| PtrbHLH35 | Potri.002G176900.1 | 491 | 54710.63 | 5.4 | 78.84 | -0.485 |
| PtrbHLH36 | Potri.002G180300.1 | 392 | 44399.54 | 6.85 | 78.09 | -0.563 |
| PtrbHLH37 | Potri.002G231700.1 | 345 | 36957.84 | 7.13 | 59.88 | -0.628 |
| PtrbHLH38 | Potri.002G235400.1 | 567 | 61701.61 | 5.63 | 60.92 | -0.713 |
| PtrbHLH39 | Potri.002G248500.1 | 563 | 61298.29 | 5.86 | 65.01 | -0.636 |
| PtrbHLH40 | Potri.002G252800.1 | 615 | 67366 | 7.29 | 55.76 | -0.671 |
| PtrbHLH41 | Potri.003G051600.1 | 334 | 36591.69 | 5.76 | 67.19 | -0.591 |
| PtrbHLH42 | Potri.003G074400.1 | 221 | 24836.14 | 5.79 | 80.27 | -0.622 |
| PtrbHLH43 | Potri.003G092200.1 | 656 | 71819.58 | 5.49 | 67.35 | -0.649 |
| PtrbHLH44 | Potri.003G093200.1 | 303 | 34006.07 | 9.08 | 91.12 | -0.259 |
| PtrbHLH45 | Potri.003G128000.1 | 647 | 72133.72 | 5.76 | 88.44 | -0.423 |
| PtrbHLH46 | Potri.003G147300.1 | 465 | 52339.91 | 7.72 | 79.2 | -0.479 |
| PtrbHLH47 | Potri.003G164500.1 | 230 | 25957.46 | 9.62 | 80.52 | -0.591 |
| PtrbHLH48 | Potri.003G207200.1 | 448 | 49944.03 | 6.83 | 52.95 | -0.934 |
| PtrbHLH49 | Potri.004G029100.1 | 482 | 52894.06 | 5.87 | 61.7 | -0.536 |
| PtrbHLH50 | Potri.004G029800.1 | 209 | 23607.37 | 6.05 | 91 | -0.338 |
| PtrbHLH51 | Potri.004G031800.1 | 243 | 27167.82 | 5.38 | 71.81 | -0.621 |
| PtrbHLH52 | Potri.004G044400.1 | 184 | 20802.45 | 6.39 | 78.37 | -0.649 |
| PtrbHLH53 | Potri.004G055700.1 | 264 | 29163.1 | 7.64 | 79.47 | -0.375 |
| PtrbHLH54 | Potri.004G088900.1 | 334 | 36654.99 | 6.02 | 64.01 | -0.585 |
| PtrbHLH55 | Potri.004G099400.1 | 276 | 31156.3 | 8.71 | 68.26 | -0.696 |
| PtrbHLH56 | Potri.004G112900.1 | 332 | 36632.43 | 5.29 | 72.29 | -0.762 |
| PtrbHLH57 | Potri.004G128400.1 | 90 | 10414.91 | 9.03 | 106.11 | -0.549 |
| PtrbHLH58 | Potri.004G156000.1 | 463 | 51471.75 | 6.48 | 68.23 | -0.526 |
| PtrbHLH59 | Potri.004G168100.1 | 326 | 36155.27 | 5.89 | 61.32 | -0.925 |
| PtrbHLH60 | Potri.005G001800.1 | 742 | 79784.41 | 5.89 | 58.77 | -0.573 |
| PtrbHLH61 | Potri.005G039800.1 | 345 | 39090.87 | 6.01 | 76.26 | -0.58 |
| PtrbHLH62 | Potri.005G053500.1 | 331 | 35169.75 | 6.09 | 79.91 | -0.292 |
| PtrbHLH63 | Potri.005G060900.1 | 189 | 21388.29 | 6.5 | 78.47 | -0.704 |
| PtrbHLH64 | Potri.005G071100.1 | 314 | 34776.02 | 7.18 | 73.92 | -0.477 |
| PtrbHLH65 | Potri.005G095400.1 | 232 | 26031.75 | 9.5 | 104.61 | -0.16 |
| PtrbHLH66 | Potri.005G113400.1 | 318 | 35288.31 | 6.34 | 62.3 | -0.819 |
| PtrbHLH67 | Potri.005G121900.1 | 271 | 30270.18 | 6.55 | 73.39 | -0.638 |
| PtrbHLH68 | Potri.005G138900.1 | 263 | 29416.92 | 8.49 | 61.56 | -0.451 |
| PtrbHLH69 | Potri.005G139700.1 | 327 | 35941.34 | 6.13 | 76.36 | -0.515 |
| PtrbHLH70 | Potri.005G146500.1 | 407 | 44242.06 | 5.36 | 66.83 | -0.621 |
| PtrbHLH71 | Potri.005G158100.1 | 365 | 40433.94 | 5.09 | 63.59 | -0.722 |
| PtrbHLH72 | Potri.005G207200.1 | 478 | 52311.36 | 9.17 | 59.81 | -0.452 |
| PtrbHLH73 | Potri.005G208600.1 | 693 | 77990.34 | 5.65 | 73.88 | -0.676 |
| PtrbHLH74 | Potri.005G217800.1 | 300 | 31778.01 | 5.75 | 77.5 | -0.311 |
| PtrbHLH75 | Potri.005G221100.1 | 504 | 55458.62 | 6.13 | 76.59 | -0.426 |
| PtrbHLH76 | Potri.005G230800.1 | 382 | 41858.54 | 7.13 | 67.72 | -0.541 |
| PtrbHLH77 | Potri.006G037100.1 | 240 | 27232.04 | 8.55 | 87.75 | -0.541 |
| PtrbHLH78 | Potri.006G037200.1 | 252 | 28481.27 | 7.89 | 88.13 | -0.479 |
| PtrbHLH79 | Potri.006G037600.1 | 250 | 28468.19 | 6 | 83.4 | -0.473 |
| PtrbHLH80 | Potri.006G057200.1 | 354 | 39105.41 | 5.51 | 71.95 | -0.662 |
| PtrbHLH81 | Potri.006G057600.1 | 355 | 39088.41 | 9.03 | 66.2 | -0.664 |
| PtrbHLH82 | Potri.006G074600.1 | 247 | 28303.65 | 7.65 | 84.53 | -0.543 |
| PtrbHLH83 | Potri.006G074700.1 | 248 | 28771.71 | 5.79 | 84.52 | -0.545 |
| PtrbHLH84 | Potri.006G074800.1 | 250 | 28574.52 | 5.95 | 95.6 | -0.501 |
| PtrbHLH85 | Potri.006G074900.1 | 248 | 28321.34 | 5.3 | 88.51 | -0.445 |
| PtrbHLH86 | Potri.006G102600.1 | 165 | 18326.24 | 6.58 | 62.06 | -0.758 |
| PtrbHLH87 | Potri.006G135600.1 | 511 | 54773.49 | 4.86 | 82.62 | -0.355 |
| PtrbHLH88 | Potri.006G148800.1 | 395 | 44309.25 | 9.32 | 90.63 | -0.459 |
| PtrbHLH89 | Potri.006G186600.1 | 449 | 47467.87 | 6.14 | 66.61 | -0.504 |
| PtrbHLH90 | Potri.006G202100.1 | 463 | 51251.08 | 5.72 | 73.89 | -0.539 |
| PtrbHLH91 | Potri.007G009400.1 | 367 | 40526.12 | 8.71 | 76.02 | -0.454 |
| PtrbHLH92 | Potri.007G010500.1 | 229 | 25449.4 | 9.39 | 87.64 | -0.39 |
| PtrbHLH93 | Potri.007G020200.1 | 388 | 43214.42 | 8.89 | 71.91 | -0.549 |
| PtrbHLH94 | Potri.007G023600.1 | 388 | 43583 | 5.66 | 76.7 | -0.587 |
| PtrbHLH95 | Potri.007G044600.1 | 239 | 27100.1 | 8.85 | 57.57 | -0.614 |
| PtrbHLH96 | Potri.007G097600.1 | 305 | 34132.46 | 7.76 | 78.66 | -0.479 |
| PtrbHLH97 | Potri.007G108000.1 | 214 | 24633.87 | 6.75 | 75.65 | -0.761 |
| PtrbHLH98 | Potri.008G052000.1 | 208 | 23804.35 | 9.92 | 79.18 | -0.63 |
| PtrbHLH99 | Potri.008G070800.1 | 259 | 28565.44 | 9.19 | 83.24 | -0.349 |
| PtrbHLH100 | Potri.008G112000.1 | 258 | 28845.24 | 5.93 | 69.15 | -0.767 |
| PtrbHLH101 | Potri.008G113200.1 | 572 | 61435.81 | 5.56 | 62.27 | -0.583 |
| PtrbHLH102 | Potri.008G116000.1 | 352 | 39285.01 | 5.87 | 80.65 | -0.634 |
| PtrbHLH103 | Potri.008G161800.1 | 301 | 34501.52 | 8.96 | 74.49 | -0.501 |
| PtrbHLH104 | Potri.008G165700.1 | 166 | 18953.82 | 9.26 | 70.72 | -0.817 |
| PtrbHLH105 | Potri.008G189600.1 | 422 | 47351.01 | 7.7 | 92.39 | -0.461 |
| PtrbHLH106 | Potri.008G190800.1 | 268 | 30289.71 | 6.37 | 60.45 | -0.937 |
| PtrbHLH107 | Potri.008G202900.1 | 206 | 23061.64 | 6.65 | 105.97 | -0.06 |
| PtrbHLH108 | Potri.009G005600.1 | 312 | 34954.24 | 4.86 | 81.28 | -0.484 |
| PtrbHLH109 | Potri.009G023800.1 | 238 | 27292.35 | 9.12 | 90.84 | -0.46 |
| PtrbHLH110 | Potri.009G064700.1 | 421 | 46278.27 | 7.15 | 57.93 | -0.748 |
| PtrbHLH111 | Potri.009G081400.1 | 337 | 37854.37 | 7.18 | 83.29 | -0.325 |
| PtrbHLH112 | Potri.009G089000.1 | 354 | 38387.03 | 4.88 | 57.4 | -0.726 |
| PtrbHLH113 | Potri.009G094300.1 | 268 | 28712.96 | 8.87 | 62.16 | -0.516 |
| PtrbHLH114 | Potri.009G117300.1 | 440 | 48496.05 | 5.83 | 66.05 | -0.677 |
| PtrbHLH115 | Potri.009G129600.1 | 323 | 35697.72 | 6.22 | 62.48 | -0.884 |
| PtrbHLH116 | Potri.009G136300.1 | 585 | 66006.47 | 5.04 | 69.97 | -0.711 |
| PtrbHLH117 | Potri.010G040000.1 | 241 | 27095.17 | 6.37 | 64.36 | -0.871 |
| PtrbHLH118 | Potri.010G041500.1 | 275 | 31089.56 | 8.34 | 82.98 | -0.472 |
| PtrbHLH119 | Potri.010G072900.1 | 221 | 24767.22 | 5.42 | 82.17 | -0.577 |
| PtrbHLH120 | Potri.010G077000.1 | 219 | 24740.22 | 8.53 | 76.16 | -0.577 |
| PtrbHLH121 | Potri.010G098900.1 | 433 | 47889.05 | 6.9 | 65.57 | -0.649 |
| PtrbHLH122 | Potri.010G130000.1 | 359 | 40049.01 | 5.92 | 80.17 | -0.637 |
| PtrbHLH123 | Potri.010G136100.1 | 562 | 60018.08 | 5.63 | 64.57 | -0.55 |
| PtrbHLH124 | Potri.010G137600.1 | 322 | 35594.5 | 5.29 | 69.72 | -0.801 |
| PtrbHLH125 | Potri.010G186700.1 | 259 | 28581.25 | 6.53 | 82.08 | -0.356 |
| PtrbHLH126 | Potri.010G208600.1 | 206 | 23181.58 | 9.39 | 85.24 | -0.55 |
| PtrbHLH127 | Potri.011G031000.1 | 243 | 26998.37 | 5.52 | 74.2 | -0.656 |
| PtrbHLH128 | Potri.011G033000.1 | 483 | 53137.26 | 5.87 | 61.8 | -0.544 |
| PtrbHLH129 | Potri.011G053400.1 | 183 | 20541.95 | 6.11 | 76.72 | -0.675 |
| PtrbHLH130 | Potri.011G065500.1 | 224 | 24786.57 | 9.53 | 87.19 | -0.398 |
| PtrbHLH131 | Potri.011G080000.1 | 239 | 26664.67 | 5.63 | 59.12 | -0.705 |
| PtrbHLH132 | Potri.011G129500.1 | 452 | 49461.27 | 6.01 | 50.69 | -0.704 |
| PtrbHLH133 | Potri.011G132400.1 | 249 | 27538.06 | 7.72 | 59.6 | -0.725 |
| PtrbHLH134 | Potri.011G157700.1 | 298 | 33662 | 4.82 | 76.24 | -0.447 |
| PtrbHLH135 | Potri.012G031800.1 | 323 | 35495.09 | 5.1 | 88.14 | -0.34 |
| PtrbHLH136 | Potri.012G055700.1 | 273 | 30481.09 | 6.18 | 60.04 | -0.591 |
| PtrbHLH137 | Potri.012G065000.1 | 600 | 65653.83 | 8.8 | 65.2 | -0.8 |
| PtrbHLH138 | Potri.012G069500.1 | 91 | 10413.83 | 9.5 | 100.77 | -0.599 |
| PtrbHLH139 | Potri.012G072700.1 | 272 | 29838.19 | 6.01 | 66.07 | -0.754 |
| PtrbHLH140 | Potri.012G079100.1 | 179 | 20382.73 | 9.39 | 97.93 | -0.244 |
| PtrbHLH141 | Potri.012G104900.1 | 359 | 40333.66 | 7.67 | 65.18 | -0.573 |
| PtrbHLH142 | Potri.012G106000.1 | 546 | 60311.48 | 5.92 | 72.49 | -0.605 |
| PtrbHLH143 | Potri.012G132000.1 | 240 | 27438.19 | 9.08 | 83.21 | -0.633 |
| PtrbHLH144 | Potri.012G132100.1 | 322 | 36332.22 | 8.38 | 86.93 | -0.422 |
| PtrbHLH145 | Potri.013G001300.1 | 719 | 77620.79 | 5.61 | 56.19 | -0.665 |
| PtrbHLH146 | Potri.013G025900.1 | 343 | 38762.85 | 6.08 | 75.6 | -0.527 |
| PtrbHLH147 | Potri.013G041000.1 | 363 | 39200.52 | 5.78 | 84.93 | -0.28 |
| PtrbHLH148 | Potri.013G107500.1 | 264 | 28486.92 | 7.6 | 63.67 | -0.706 |
| PtrbHLH149 | Potri.013G117600.1 | 268 | 29325.65 | 8.41 | 90.3 | -0.231 |
| PtrbHLH150 | Potri.013G126800.1 | 143 | 15828.17 | 9.22 | 76.43 | -0.39 |
| PtrbHLH151 | Potri.013G129800.1 | 218 | 24556 | 5.27 | 88.99 | -0.445 |
| PtrbHLH152 | Potri.014G017100.1 | 343 | 38073.41 | 5.01 | 61.75 | -0.666 |
| PtrbHLH153 | Potri.014G025800.1 | 310 | 33741.64 | 5.42 | 71.13 | -0.54 |
| PtrbHLH154 | Potri.014G025900.1 | 291 | 32121.52 | 8.48 | 75.77 | -0.346 |
| PtrbHLH155 | Potri.014G027300.1 | 265 | 29538.4 | 8.87 | 64.11 | -0.611 |
| PtrbHLH156 | Potri.014G066500.1 | 471 | 51139.76 | 8.43 | 54.54 | -0.585 |
| PtrbHLH157 | Potri.014G099700.1 | 619 | 68046.23 | 6.16 | 70.78 | -0.527 |
| PtrbHLH158 | Potri.014G103700.1 | 493 | 54887.76 | 5.91 | 79.86 | -0.505 |
| PtrbHLH159 | Potri.014G106300.1 | 390 | 44137.83 | 5.4 | 79.74 | -0.559 |
| PtrbHLH160 | Potri.014G111400.1 | 561 | 61476.18 | 6.97 | 65.06 | -0.621 |
| PtrbHLH161 | Potri.014G148900.1 | 572 | 62308.33 | 7.98 | 61.92 | -0.751 |
| PtrbHLH162 | Potri.014G150600.1 | 342 | 36344.26 | 8.73 | 62.75 | -0.614 |
| PtrbHLH163 | Potri.015G022300.1 | 303 | 33154.35 | 4.72 | 91.68 | -0.239 |
| PtrbHLH164 | Potri.015G046300.1 | 274 | 31040.79 | 6.62 | 58.76 | -0.647 |
| PtrbHLH165 | Potri.015G048000.1 | 567 | 61793.56 | 8.45 | 64.71 | -0.767 |
| PtrbHLH166 | Potri.015G063300.1 | 91 | 10430.78 | 8.97 | 92.2 | -0.766 |
| PtrbHLH167 | Potri.015G068100.1 | 274 | 30158.35 | 5.82 | 59.85 | -0.846 |
| PtrbHLH168 | Potri.015G074500.1 | 178 | 20393.67 | 9.27 | 91.85 | -0.251 |
| PtrbHLH169 | Potri.015G104200.1 | 357 | 40215.16 | 7.23 | 61.48 | -0.713 |
| PtrbHLH170 | Potri.015G105200.1 | 558 | 61145.47 | 5.88 | 76.18 | -0.557 |
| PtrbHLH171 | Potri.015G134300.1 | 246 | 27754.52 | 8.29 | 84.8 | -0.552 |
| PtrbHLH172 | Potri.015G134400.1 | 247 | 27845.05 | 5.68 | 86.8 | -0.629 |
| PtrbHLH173 | Potri.015G142700.1 | 241 | 26541.06 | 6.25 | 82.16 | -0.562 |
| PtrbHLH174 | Potri.016G035400.1 | 241 | 27187 | 8.54 | 90.25 | -0.486 |
| PtrbHLH175 | Potri.016G037300.1 | 264 | 29974.78 | 6.72 | 84.92 | -0.486 |
| PtrbHLH176 | Potri.016G050500.1 | 351 | 38904.03 | 9.28 | 61.71 | -0.803 |
| PtrbHLH177 | Potri.016G051100.1 | 348 | 38734.18 | 5.79 | 72.36 | -0.705 |
| PtrbHLH178 | Potri.016G068500.1 | 467 | 52090.04 | 5.32 | 74.33 | -0.534 |
| PtrbHLH179 | Potri.016G120800.1 | 163 | 18127.39 | 6.9 | 70.55 | -0.59 |
| PtrbHLH180 | Potri.017G041000.1 | 315 | 34513.41 | 4.98 | 65.65 | -0.376 |
| PtrbHLH181 | Potri.017G054500.1 | 421 | 47981.93 | 5.22 | 77.36 | -0.61 |
| PtrbHLH182 | Potri.017G081300.1 | 90 | 10287.76 | 7.94 | 108.33 | -0.439 |
| PtrbHLH183 | Potri.017G101700.1 | 332 | 36780.66 | 5.7 | 71.99 | -0.758 |
| PtrbHLH184 | Potri.017G115300.1 | 265 | 30035.57 | 6.22 | 63.36 | -0.792 |
| PtrbHLH185 | Potri.017G126800.1 | 330 | 36197.27 | 6.11 | 63.91 | -0.7 |
| PtrbHLH186 | Potri.018G083700.1 | 350 | 37922.32 | 6.31 | 66.06 | -0.532 |
| PtrbHLH187 | Potri.018G109500.1 | 455 | 47865.21 | 5.8 | 67.89 | -0.485 |
| PtrbHLH188 | Potri.018G141500.1 | 274 | 31688.12 | 5.97 | 82.59 | -0.605 |
| PtrbHLH189 | Potri.018G141600.1 | 216 | 24906.15 | 5.21 | 78.1 | -0.614 |
| PtrbHLH190 | Potri.018G141700.1 | 247 | 29002.12 | 5.94 | 92.39 | -0.549 |
| PtrbHLH191 | Potri.018G141800.1 | 249 | 28175.14 | 5.16 | 88.55 | -0.426 |
| PtrbHLH192 | Potri.019G034700.1 | 242 | 27263.93 | 6.19 | 82.19 | -0.379 |
| PtrbHLH193 | Potri.019G079900.1 | 246 | 26797.84 | 6.03 | 61.54 | -0.724 |
| PtrbHLH194 | Potri.019G089000.1 | 267 | 29402.62 | 8.25 | 88.46 | -0.316 |
| PtrbHLH195 | Potri.019G089300.1 | 85 | 9642.11 | 9.39 | 90.47 | -0.409 |
| PtrbHLH196 | Potri.019G099300.1 | 216 | 24354.52 | 9.16 | 79.49 | -0.498 |
| PtrbHLH197 | Potri.019G099400.1 | 222 | 25035.26 | 8.97 | 76.44 | -0.527 |
| PtrbHLH198 | Potri.019G099500.1 | 232 | 26157.6 | 7.8 | 81.55 | -0.439 |
| PtrbHLH199 | Potri.019G112000.1 | 143 | 15721.06 | 9.22 | 79.16 | -0.355 |
| PtrbHLH200 | Potri.T107900.1 | 198 | 23046.2 | 6.05 | 80.2 | -0.63 |
| PtrbHLH201 | Potri.T155900.1 | 312 | 34970.24 | 4.86 | 81.28 | -0.497 |
| PtrbHLH202 | Potri.T179100.1 | 60 | 6917.34 | 6.09 | 32.67 | -1.885 |
