# Supplementary material for: Characterization of the basic helix–loop–helix gene family and its tissue-differential expression in response to salt stress in poplar
Source: PeerJ. 2018 Mar 14;6:e4502. doi: 10.7717/peerj.4502 (PMC5857177; doi:10.7717/peerj.4502)
Supplement: Supplemental Information 12 [file peerj-06-4502-s012.docx]

The DNA binding domain alignment of poplar bHLH family

| Gene ID/Start-End | DNA binding domain alignment |
| --- | --- |

Potri.002G054100.1/479-524 --------------------------------NHVLAERRRREKLNERFI

Potri.005G208600.1/473-519 --------------------------------NHVLAERRRREKLNERFI

Potri.002G172100.1/375-421 --------------------------------NHVEAERQRREKLNQRFY

Potri.014G099700.1/450-496 --------------------------------NHVEAERQRREKLNQRFY

Potri.002G042000.1/358-404 --------------------------------NHVEAERQRREKLNQRFY

Potri.005G221100.1/359-405 --------------------------------NHVEAERQRREKLNQRFY

Potri.003G092200.1/480-525 --------------------------------NHVEAERQRREKLNQRFY

Potri.001G142200.1/471-516 --------------------------------NHVEAERQRREKLNQRFY

Potri.002G176900.1/317-362 --------------------------------NHVEAERQRREKLNHRFY

Potri.014G103700.1/317-362 --------------------------------NHVEAERQRREKLNHRFY

Potri.003G147300.1/289-334 --------------------------------NHVEAERQRRERLNHRFY

Potri.001G083500.1/295-340 --------------------------------NHVEAERQRRERLNHRFY

Potri.009G081400.1/159-206 -------------------------------QDHIIAERKRREKLSQRFI

Potri.001G287200.1/152-199 -------------------------------QDHIIAERKRREKLSQRFI

Potri.005G095400.1/57-103 --------------------------------SHAIEERNRREKLSQRFI

Potri.007G009400.1/180-226 --------------------------------DHIMAERKRREKLSQQFI

Potri.017G101700.1/43-92 ------------------------------RSRHSETEQRRRSKINERFQ

Potri.T179100.1/43-60 ------------------------------RSRHSETEQRRRSKINER--

Potri.004G112900.1/43-92 ------------------------------RSKHSETEQRRRSKINERFQ

Potri.010G137600.1/51-100 ------------------------------RSKHSVTEQKRRSKINERFQ

Potri.008G112000.1/51-100 ------------------------------RSKHSVTEQRRRSKINERFQ

Potri.015G048000.1/263-312 ------------------------------RSKHSATEQRRRSKINDRFQ

Potri.012G065000.1/294-326 -----------------------------------------------RFQ

Potri.002G143300.1/297-343 --------------------------------VHNQSERKRRDKINQRMK

Potri.014G066500.1/297-342 ---------------------------------HNQSERKRRDKINQRMK

Potri.014G025800.1/127-173 --------------------------------VHNLSEKRRRSRINEKMK

Potri.005G139700.1/143-188 ---------------------------------HNLSEKRRRSRINEKMK

Potri.002G124400.1/150-170 ------------------------------------SEK-----------

Potri.005G207200.1/272-318 --------------------------------VHNLSERRRRDRINEKMR

Potri.002G055400.1/388-434 --------------------------------VHNLSERRRRDRINEKMR

Potri.002G252800.1/371-417 --------------------------------VHNLSERRRRDRINEKMR

Potri.013G001300.1/455-501 --------------------------------VHNLSERRRRDRINEKMR

Potri.005G001800.1/470-516 --------------------------------VHNLSERRRRDRINEKMR

Potri.014G111400.1/386-431 ---------------------------------HNLSERKRRDRINKKMR

Potri.013G107500.1/200-245 ----------------------------------SIAERVRRTRISDRIR

Potri.019G079900.1/182-227 ----------------------------------SIAERVRRTRISDRIR

Potri.016G050500.1/279-324 ----------------------------------SIAERVRRTRISERMR

Potri.006G057600.1/283-328 ----------------------------------SIAERVRRTRISERMR

Potri.009G064700.1/349-394 ----------------------------------SIAERVRRTRISERMR

Potri.003G207200.1/382-427 ----------------------------------SIAERVRRTRISERMR

Potri.001G270000.1/348-393 ----------------------------------SIAERVRRTRISERMR

Potri.014G150600.1/275-320 ----------------------------------SIAERERRTRISGKLK

Potri.002G231700.1/278-323 ----------------------------------SIAERERRTRISGKLK

Potri.006G186600.1/251-296 ---------------------------------HSIAERLRRERIAERMK

Potri.018G109500.1/252-297 ---------------------------------HSIAERLRRERIAERMK

Potri.006G135600.1/306-351 ---------------------------------HSIAERLRREKIAERMK

Potri.005G217800.1/149-193 ---------------------------------HSIAERLRRERIAERIR

Potri.002G045400.1/149-193 ---------------------------------HSIAERLRRERIAERIR

Potri.013G041000.1/181-225 ---------------------------------HSIAERLRRVRITERVK

Potri.005G053500.1/180-224 ---------------------------------HSIAERLRRVRITERVK

Potri.010G040000.1/125-172 --------------------------------SHSLAERARREKISERMK

Potri.015G068100.1/155-202 --------------------------------SHSLAERARREKISERMN

Potri.008G190800.1/151-198 --------------------------------SHSLAERARREKISERMK

Potri.012G072700.1/153-200 --------------------------------SHSLAERARRERIGERMK

Potri.002G114700.1/241-289 -------------------------------NSHSLAERVRREKISERMR

Potri.005G146500.1/249-297 -------------------------------NSHSLAERVRREKISERMR

Potri.009G117300.1/247-294 --------------------------------SHSLAERVRREKISERMK

Potri.004G156000.1/277-318 --------------------------------------QVRREKISERMK

Potri.002G248500.1/368-415 --------------------------------SHSLAERVRREKISERMK

Potri.002G100100.1/142-189 --------------------------------SHSLVERVRREKISERMK

Potri.010G136100.1/378-426 -------------------------------NSHSLAERVRREKISERMK

Potri.008G113200.1/383-431 -------------------------------NSHSLAERVRREKISERMK

Potri.014G148900.1/379-426 --------------------------------SHSLAERVRREKISERMK

Potri.002G235400.1/379-426 --------------------------------SHSLAERVRREKISERMK

Potri.015G104200.1/184-231 --------------------------------SHSLAERVRRERISERMK

Potri.012G104900.1/182-229 --------------------------------SHSLAERVRREKISERMK

Potri.005G121900.1/182-229 --------------------------------SHSLAERARREKISKKMK

Potri.007G023600.1/228-275 --------------------------------SHSLAERARREKISKKMK

Potri.006G057200.1/187-234 --------------------------------SHSLAERARREKINQRMK

Potri.016G051100.1/192-239 --------------------------------SHSLAERARREKINQRMK

Potri.001G062900.1/87-133 --------------------------------SHSLAERVRRERISAKMK

Potri.003G164500.1/171-217 --------------------------------SHSLAERVRREKISSRMK

Potri.004G099400.1/168-215 --------------------------------SHSIAERIRREKINNKLR

Potri.017G115300.1/158-205 --------------------------------SHSIAERVRREKINNKLR

Potri.015G046300.1/167-214 --------------------------------SHSLAERVRRGKINERLR

Potri.012G055700.1/166-213 --------------------------------SHSLAERVRRGKINERLR

Potri.009G089000.1/280-320 --------------------------------------RKRRERINERLR

Potri.001G294300.1/240-280 --------------------------------------RKRRERINERLR

Potri.014G017100.1/286-326 --------------------------------------RKRRERINERLK

Potri.002G119200.1/288-328 --------------------------------------RKRRERINERLK

Potri.004G088900.1/244-287 -----------------------------------IAAKNRRERISERLK

Potri.017G126800.1/240-283 -----------------------------------IAAKNRRERISERLK

Potri.006G102600.1/46-86 -------------------------------------ARERRHRISDRFK

Potri.016G120800.1/46-86 -------------------------------------ARERRHRISDRFK

Potri.T107900.1/122-162 -------------------------------------ARLRRERISEKIR

Potri.007G108000.1/122-162 -------------------------------------ARLRRERISEKIR

Potri.005G060900.1/77-117 -------------------------------------ARLRRERISEKIR

Potri.002G125000.1/165-205 -------------------------------------ARHRRERISERIR

Potri.005G138900.1/171-211 -------------------------------------ARHRRERISERIR

Potri.014G027300.1/167-207 -------------------------------------ARHRRERISERIR

Potri.007G044600.1/172-212 -------------------------------------ARHRRERISERMR

Potri.001G191800.1/347-387 -------------------------------------ARQRRERISDRIR

Potri.001G461000.1/173-217 ----------------------------------SIAARERRRKITEKTQ

Potri.011G157700.1/174-218 ----------------------------------SIAARERRRKITEKTR

Potri.008G052000.1/98-137 ---------------------------------------RRSQKLSDKIT

Potri.010G208600.1/111-150 ---------------------------------------RRSQKLSDKIT

Potri.001G410600.1/317-355 ---------------------------------------VRKEKMGDRIT

Potri.011G129500.1/343-381 ---------------------------------------ARKEKMGDRIT

Potri.004G029100.1/357-394 ----------------------------------------RKEKLGDRIT

Potri.011G033000.1/358-395 ----------------------------------------RKEKLGDRIT

Potri.003G051600.1/193-231 ----------------------------------------RKEKLGDRIT

Potri.001G185900.1/ ---------------------------------------VRKEKLGDRIT

Potri.018G083700.1/216-254 ----------------------------------------RKEKLGDRIT

Potri.005G230800.1/319-357 ----------------------------------------RKEKLGDRIA

Potri.002G032400.1/319-358 ---------------------------------------ARKEKLGDRIA

Potri.007G020200.1/288-325 ----------------------------------------RKEKLGDRIA

Potri.013G126800.1/33-72 --------------------------------------KERKEKLGERIV

Potri.019G112000.1/33-72 --------------------------------------KERKEKLGERIV

Potri.001G299300.1/160-197 -----------------------------------------REKLGDRIA

Potri.009G094300.1/160-197 -----------------------------------------REKVGDRIT

Potri.010G098900.1/342-361 --------------------------------------------------

Potri.005G158100.1/314-353 --------------------------------------RMRKENIRETMS

Potri.017G041000.1/221-257 -------------------------------------------RIAERVK

Potri.011G080000.1/189-225 -----------------------------------------RQKMKTMVK

Potri.006G037100.1/70-116 -------------------------------KNHKEAEKRRRERINSHLD

Potri.016G035400.1/71-117 -------------------------------KNHKEAEKRRRERINSHLD

Potri.002G105300.1/25-72 ------------------------------CKSHKETERRRRQRINAHLS

Potri.010G130000.1/165-211 -------------------------------KSHSEAERRRRERINNHLA

Potri.008G116000.1/163-209 -------------------------------KSHSEAERRRRERINNHLA

Potri.004G055700.1/75-117 -------------------------------KSHSEAERRRRERINAHLD

Potri.011G065500.1/39-80 -------------------------------KSHSEAERRRRERINAHLA

Potri.013G117600.1/74-120 -------------------------------RNHSEAERKRRARINAHLD

Potri.019G089000.1/74-120 -------------------------------KNHSEAEKKRRARINAHLD

Potri.019G089300.1/47-70 ----------------------------------SEAERKPRAKINKHLD

Potri.008G070800.1/69-115 -------------------------------KSHSQAEKRRRDRINAQLG

Potri.010G186700.1/69-115 -------------------------------KSHSQAEKRRRDRINAQLG

Potri.007G010500.1/61-107 -------------------------------KKHSMAESNRRSRINTQFT

Potri.T155900.1/132-175 -----------------------------------ISERRRRGRMKEKLY

Potri.009G005600.1/132-175 -----------------------------------ISERRRRGRMKEKLY

Potri.008G161800.1/159-207 ------------------------------CYRHKMSERLRRQRERNGYL

Potri.010G077000.1/74-123 -----------------------------RCNRHMMSERLRRERERHGYL

Potri.004G029800.1/41-85 ---------------------------------NLHAERRRREKLSNRLL

Potri.018G141500.1/54-99 ---------------------------------NTVSERNRRKKLNDKLL

Potri.006G074600.1/57-99 ------------------------------------SERKRRKKLNDKLL

Potri.018G141600.1/55-99 ----------------------------------TVSERNRRKKLNDKLY

Potri.006G074800.1/52-100 ------------------------------RTKNIALETNKRKELNDKLL

Potri.006G074700.1/59-102 -----------------------------------VSERSRRKKLSDKLL

Potri.018G141700.1/57-100 -----------------------------------VSERSRRQKLSDKLL

Potri.006G074900.1/57-99 -----------------------------------VSERNRRKRLNERLF

Potri.018G141800.1/56-99 ----------------------------------IVSERNRRKKLNERLF

Potri.008G189600.1/234-276 -----------------------------------VTERNRRTRIKTGLF

Potri.010G041500.1/115-157 -----------------------------------VTERNRRTRIKTGLF

Potri.015G105200.1/374-416 -----------------------------------MAERRRRKKLNDRLY

Potri.012G106000.1/362-404 -----------------------------------MAERRRRKKLNDRLY

Potri.002G108400.1/181-224 ----------------------------------LMAERRRRKRLNDRLS

Potri.009G136300.1/336-379 -----------------------------------VAERKRRKKLNDRLY

Potri.012G031800.1/89-140 -----------------------------QRMSHITVERNRRKQMNEHLS

Potri.015G022300.1/96-147 -----------------------------QRISHITVERNRRKQMNEHLS

Potri.008G202900.1/2-49 --------------------------------SHIAVERNRRRQMNEHLK

Potri.013G025900.1/140-191 -----------------------------QRMTHIAVERNRRKQMNEYLS

Potri.005G039800.1/142-193 -----------------------------QRMTHIAVERNRRKQMNEYLS

Potri.002G101900.1/128-179 -----------------------------QRMTHIAVERNRRKQMNEYLA

Potri.005G071100.1/101-152 -----------------------------QRMTHIKVERNRRKQMNDYLT

Potri.007G097600.1/89-140 -----------------------------QRMTHINVERNRRKQMNEYLA

Potri.003G093200.1/99-149 -----------------------------QRMTHIAVERNRRKLMNEHLA

Potri.001G141100.1/99-150 -----------------------------QRMTHIAVERNRRKLMNGYLA

Potri.001G314400.1/210-261 -----------------------------QRMTHIAVERNRRKQMNEHLR

Potri.017G054500.1/223-274 -----------------------------QRMTHIAVERNRRKQMNEHLR

Potri.002G180300.1/200-251 -----------------------------QRMNHIAVERKRRRLMNDHLN

Potri.014G106300.1/198-249 -----------------------------QRMNHIAVERNRRRLMNDHLN

Potri.016G068500.1/260-306 -------------------------------TKHFATERQRREHLNGKYT

Potri.006G202100.1/259-305 -------------------------------TKHFATERQRREHLNGKYT

Potri.009G023800.1/42-86 ----------------------------------FAIELQRRGQLNDNYK

Potri.006G037600.1/70-122 -----------------------------KKLNHNASERDRRKKINSLYS

Potri.016G037300.1/71-123 -----------------------------KKLNHNASERDRRKKINSLYS

Potri.006G037200.1/72-123 -----------------------------KKLSHNANERDRRKKIKSLYS

Potri.015G134300.1/72-124 -----------------------------KKMMHRDIERQRRQEMTTLYA

Potri.012G132000.1/72-124 -----------------------------KKMMHRNIERQRRQEMTTLYA

Potri.001G113400.1/80-129 -------------------------------IIHRDIERQRRQEMANLYG

Potri.015G134400.1/75-127 -----------------------------KKVARKEIERQRRQQMSTLHA

Potri.012G132100.1/153-204 -----------------------------KKIARKEIERQRRQHISTLHA

Potri.011G053400.1/9-61 ------------------------------RTDRKTIERNRRNQMKALYS

Potri.001G063000.1/9-61 ------------------------------RTDRKTIERNRRNQMKALYS

Potri.004G044400.1/9-61 ------------------------------RTDRKLIERNRRNQMKELYS

Potri.019G099300.1/24-74 -------------------------------TERKVVERNRRNQMKSLYS

Potri.019G099400.1/31-81 -------------------------------TERKVVERNRRNQMKSLYS

Potri.019G099500.1/40-90 -------------------------------TERKVVERNRRNQMKSLYS

Potri.013G129800.1/22-72 -------------------------------TERKIIEKNRRNQMKTLYS

Potri.019G034700.1/67-116 --------------------------------ERKVSEKNRRNQMKTLYS

Potri.012G079100.1/13-64 ---------------------------------RKTVERNRRIHMKDLCL

Potri.015G074500.1/14-63 ----------------------------------KTVEKNRRVHMKDLCF

Potri.012G069500.1/22-59 ---------------------------------------------LDLVT

Potri.015G063300.1/22-59 ---------------------------------------------LDLVT

Potri.017G081300.1/18-57 -------------------------------------------QIIDLVS

Potri.004G128400.1/23-57 ------------------------------------------------VS

Potri.001G305100.1/89-136 --------------------------------THIWTERERRKKMRTMFS

Potri.014G025900.1/84-127 ------------------------------------TERERRKKMRNMFS

Potri.003G128000.1/446-492 --------------------------------SHVLSERRRREKLNKRFM

Potri.001G103600.1/431-478 --------------------------------SHALSERKQREKLNKRFM

Potri.002G159400.1/465-501 ------------------------------------------QRVNDKFI

Potri.008G165700.1/15-62 --------------------------------TKACREKLRRDRLNDRFQ

Potri.010G072900.1/70-117 --------------------------------TKACREKLRRERLNDRFQ

Potri.001G416600.1/107-154 --------------------------------SKACREKLRRDRLNDKFI

Potri.011G132400.1/92-139 --------------------------------SKACREKLRRDRLNDKFM

Potri.011G031000.1/87-133 ---------------------------------KACREKMRRDRLNDRFT

Potri.004G031800.1/87-133 ---------------------------------KACREKMRRDRLNDRFM

Potri.009G129600.1/40-87 -------------------------------IQKADREKLRRDRLNEHFV

Potri.004G168100.1/44-91 -------------------------------IQKADREKLRRDRLNEHFV

Potri.005G113400.1/62-110 ------------------------------KVQKADREKLRRDNLNEQFL

Potri.003G074400.1/31-80 -----------------------------KRIHKSEREKLKREQLNELFL

Potri.015G142700.1/31-80 -----------------------------RRIHKAEREKLKREQLNELFL

Potri.006G148800.1/221-265 ---------------------------------HMISERKRREKINESFK

Potri.002G054100.1/479-524 ILRSLVPF-V-------TK--MDKASILGDTIEYVKQL------

Potri.005G208600.1/473-519 MLRSLVPF-V-------TK--MDKASILGDTIEYVKQLR-----

Potri.002G172100.1/375-421 ALRAVVPNIS--------K--MDKASLLGDAISYINELQ-----

Potri.014G099700.1/450-496 ALRAVVPNIS--------K--MDKASLLGDAISYINELQ-----

Potri.002G042000.1/358-404 ALRAVVPNIS--------K--MDKASLLGDAITYITDLQ-----

Potri.005G221100.1/359-405 ALRAVVPNIS--------K--MDKASLLGDAITFITDLQ-----

Potri.003G092200.1/480-525 ALRAVVPN-V-------SK--MDKASLLGDAISYINEL------

Potri.001G142200.1/471-516 ALRAVVPN-V-------SK--MDKASLLGDAISYIDEL------

Potri.002G176900.1/317-362 ALRAVVPN-V-------SR--MDKASLLSDAVSYINEL------

Potri.014G103700.1/317-362 ALRAVVPN-V-------SR--MDKASLLSDAVSYINEM------

Potri.003G147300.1/289-334 ALRSVVPN-V-------SK--MDRASLLADAVNYIKEL------

Potri.001G083500.1/295-340 ALRSVVPN-V-------SK--MDKASLLADAATYIKEL------

Potri.009G081400.1/159-206 ALSAVVPG-L-------KK--MDKASVLGDAIKYLKQLQ-----

Potri.001G287200.1/152-199 ALSAVVPG-L-------KK--MDKASVLGDAIKYLKQLQ-----

Potri.005G095400.1/57-103 ALSAVVPG-L-------KK--MDKASVLGDAIKYLKYLQ-----

Potri.007G009400.1/180-226 ALSALVPG-L-------KK--MDKASVLDGAMKYMKQLQ-----

Potri.017G101700.1/43-92 ALRNLVPQ-N------DQK--RDKASFLLEVIEYVQFLQ-----

Potri.T179100.1/43-60 --------------------------------------------

Potri.004G112900.1/43-92 ALRNLIPQ-N------DQK--RDKASFLLEVIEYIQFLQ-----

Potri.010G137600.1/51-100 ILRDLIPH-S------DQK--RDTASFLLEVIEYVQYLQ-----

Potri.008G112000.1/51-100 ILRDLIPHS-------DQK--RDTASFLLEVIEYVQHLQ-----

Potri.015G048000.1/263-312 MLRALIPH-G------DQK--RDKASFLLEVIEHVQFLQ-----

Potri.012G065000.1/294-326 MLRELIPR-G------DQK--KDKASFLLEVIEYIQFLQ-----

Potri.002G143300.1/297-343 TLQKLVPN-S-------SK--TDKASMLDEVIEYLKQLQ-----

Potri.014G066500.1/297-342 TLQKLVPS-S-------SK--TDKASMLDEVIEYLKQLQ-----

Potri.014G025800.1/127-173 ALQNLIPN-S-------NK--TDKASMLDEAIEYLKQLQ-----

Potri.005G139700.1/143-188 ALQNLIPN-S-------SK--TDKASMLDEAIEYLKLLQ-----

Potri.002G124400.1/150-170 ---------------------TDKASMLDEAIEYLKQLQ-----

Potri.005G207200.1/272-318 ALQELIPH-C-------YK--TDKASMLDEAIEYLKSLQ-----

Potri.002G055400.1/388-434 ALQELIPH-C-------NK--TDKASMLDEAIEYLKSLQ-----

Potri.002G252800.1/371-417 ALQELIPR-C-------NK--SDKASMLDEAIEYLKSLQ-----

Potri.013G001300.1/455-501 ALQELIPN-C-------NK--VDKASMLDEAIEYLKTLQ-----

Potri.005G001800.1/470-516 ALQELIPN-C-------NK--VDKASMLDEAIEYLKTLQ-----

Potri.014G111400.1/386-431 ALQDLIPN-S-------NK--VDKASMLGEAIDYLKSLQ-----

Potri.013G107500.1/200-245 KLQELVPN-M-------DKQ-TNTADMLEEAVDYVKFLQ-----

Potri.019G079900.1/182-227 KLQELVPN-M-------DKQ-TNTADMLDEALAYVKFLQ-----

Potri.016G050500.1/279-324 KLQELVPN-M-------DKQ-TNTADMLDLAVDYIKDLQ-----

Potri.006G057600.1/283-328 KLQELVPN-M-------DKQ-TNTADMLDLAVVYIKDLQ-----

Potri.009G064700.1/349-394 KLQELFPN-M-------DKQ-TNTADMLDLAVEHIKDLQ-----

Potri.003G207200.1/382-427 KLQDLVPN-M-------DKQ-TNTSDMLDLAVDYIKDLQ-----

Potri.001G270000.1/348-393 KLQELFPD-M-------DKQ-TSTADKLDLSIELIKDLQ-----

Potri.014G150600.1/275-320 TLQDLVPN-M-------DKQ-TSYADMLELAVKHIKGLQ-----

Potri.002G231700.1/278-323 KLQDLVPN-M-------DKQ-TSYADMLDFAVQHIKGLQ-----

Potri.006G186600.1/251-296 ALQELVPN-A-------NK--TDKASMLDEIIDYVKFLQ-----

Potri.018G109500.1/252-297 ALQELVPN-A-------NK--TDKASMLDEIIDYVKFLQ-----

Potri.006G135600.1/306-351 NLQELVPN-S-------NK--VDKASMLDEIIEYVKFLQ-----

Potri.005G217800.1/149-193 ALQELVPSVN--------K--TDRATMLDEIVDYVKFL------

Potri.002G045400.1/149-193 ALQELVPSVN--------K--TDRAAMLDEIVDYVKFL------

Potri.013G041000.1/181-225 ALQELVPT-C-------NK--TDRAAMLDEIVDYVKFL------

Potri.005G053500.1/180-224 ALQELVPT-C-------NK--TDRAAMLDEIVDYVKFL------

Potri.010G040000.1/125-172 ILQDLVPG-C-------NKV-IGKALVLDEIINYIQSLQ-----

Potri.015G068100.1/155-202 MLQDLVPG-C-------NKV-IGKALVLDEIINYIQSLQ-----

Potri.008G190800.1/151-198 ILQDIVPG-C-------NKV-TGKALVLDEIINYIQSLQ-----

Potri.012G072700.1/153-200 ILQDLVPG-C-------NKV-IGKALALDEIINYIQSLQ-----

Potri.002G114700.1/241-289 MLQELVPG-C-------NKI-TGKAVMLDEIINYVQSLQ-----

Potri.005G146500.1/249-297 MLQELVPG-C-------NKI-TGKAVMLDEIINYVQSLQ-----

Potri.009G117300.1/247-294 YLQDLVPG-C-------NKI-TGKAGMLDEIINYVQSLQ-----

Potri.004G156000.1/277-318 YLQDLVPG-C-------NNI-TGKAGMLDEIINYVQSLQ-----

Potri.002G248500.1/368-415 LLQDLVPG-C-------NKV-TGKALMLDEIINYVQSLQ-----

Potri.002G100100.1/142-189 LLQDLVPG-C-------NKV-TGKAFMLDEIINYVQSLQ-----

Potri.010G136100.1/378-426 FLQDLVPG-C-------SKV-TGKAVMLDEIINYVQSLQ-----

Potri.008G113200.1/383-431 FLQDLVPG-C-------SKV-TGKAVMLDEIINYVQSLQ-----

Potri.014G148900.1/379-426 FLQDLVPG-C-------NKV-TGKAVMLDEIINYVQSLQ-----

Potri.002G235400.1/379-426 FLQDLVPG-C-------NKV-TGKAVMLDEIINYVQSLQ-----

Potri.015G104200.1/184-231 ILQLLVPG-C-------DKI-TGKALMLDEIINYVQSLQ-----

Potri.012G104900.1/182-229 MLQRLVPG-C-------DKV-TGKALMLDEIINYVQSLQ-----

Potri.005G121900.1/182-229 SLQDLVPG-C-------NKI-TGRAGMLDEIINYVQSLQ-----

Potri.007G023600.1/228-275 CLQDLVPG-C-------NKI-TGRAGMLDEIINYVQSLQ-----

Potri.006G057200.1/187-234 LLQELVPG-C-------NKI-SGTALVLDEIINHVQSLQ-----

Potri.016G051100.1/192-239 LLQELVPG-C-------NKI-SGTALVLDEIINHVQFLQ-----

Potri.001G062900.1/87-133 LLQSLVPG-C-------DQI-TGKALILDEIIRYVQSL------

Potri.003G164500.1/171-217 LLQSLVPG-C-------DKI-TGKALVLDEIISYVQFL------

Potri.004G099400.1/168-215 CLQDIVPG-C-------HKS-MGMAVMLEEIINYVHSLQ-----

Potri.017G115300.1/158-205 CLQDLVPG-C-------HRS-MGMAVMLEEIINYVHSLQ-----

Potri.015G046300.1/167-214 CLQDIVPG-C-------YKT-MGMAVMLDEIINYVQSLQ-----

Potri.012G055700.1/166-213 CLQDIVPG-C-------YKT-MGMAVMLDEIINYVQSLQ-----

Potri.009G089000.1/280-320 ILQTLVPN-G-------TK--VDISTMLEEAVQYVKFLQ-----

Potri.001G294300.1/240-280 ILQNLVPN-G-------TK--VDISTMLEEAVQYVKFLQ-----

Potri.014G017100.1/286-326 ILQHIVPN-G-------TK--VDISTMLEEAVHYVKFLQ-----

Potri.002G119200.1/288-328 ILQNLVPN-G-------TK--VDISTMLEEAVHYVNFLQ-----

Potri.004G088900.1/244-287 VLQDLVPN-G-------SK--VDLVTMLEKAISYVKFLQ-----

Potri.017G126800.1/240-283 MLQDLVPN-G-------SK--VDLVTMLEKAISYVKFLQ-----

Potri.006G102600.1/46-86 ILQSLVPG-G-------TK--MDTVSMLEEAINYVKFL------

Potri.016G120800.1/46-86 ILQSLVPG-G-------TK--MDTVSMLEEAINYVKFL------

Potri.T107900.1/122-162 ILQRLVPG-G-------RK--MDTASMLDEAIRYVKFL------

Potri.007G108000.1/122-162 ILQRLVPG-G-------RK--MDTASMLDEAIRYVKFL------

Potri.005G060900.1/77-117 ILQRLVPG-G-------RK--MDTASMLEEAIRYVKFL------

Potri.002G125000.1/165-205 ILQRLVPG-G-------TK--MDTASMLDEAIHYVKFL------

Potri.005G138900.1/171-211 ILQRLVPG-G-------TK--MDTASMLDEAIHYVKFL------

Potri.014G027300.1/167-207 ILQRLVPG-G-------TK--MDTASMLDEAIHYVKFL------

Potri.007G044600.1/172-212 ILQRLVPG-G-------TK--MDTASMLDEAIHYVKFL------

Potri.001G191800.1/347-387 VLQGMVPG-G-------SK--MDTASMLDEAANYLKFL------

Potri.001G461000.1/173-217 QLGKLIPGGN--------K--MNTAEMFQAASKYVKFLQ-----

Potri.011G157700.1/174-218 ELGKFIPGGH--------K--MNTAEMFQAASKYVKFLQ-----

Potri.008G052000.1/98-137 ALQKLVSP-Y-------GK--TDTASVLQEASLYIKLLQ-----

Potri.010G208600.1/111-150 ALQKLVSP-Y-------GK--ADTASVLLEASLHIKLLQ-----

Potri.001G410600.1/317-355 ALQQLVSP-F-------GK--TDTASVLSEAIEYIKFL------

Potri.011G129500.1/343-381 ALQQLVSP-F-------GK--TDTASVLSEAIEYIKFL------

Potri.004G029100.1/357-394 ALQQLVSP-F-------GK--TDTASVLHEAIEYIKFL------

Potri.011G033000.1/358-395 ALQQLVSP-F-------GK--TDTASVLHEAIEYIKFL------

Potri.003G051600.1/193-231 ALHQMVSP-F-------GK--TDTASVLLEAIGYIRFLQ-----

Potri.001G185900.1/ VLHQMVSP-F-------GK--TDTASVLLEAIGYIRFLQGQIEA

Potri.018G083700.1/216-254 ALHQLVSP-F-------GK--TDTASVLLEAIGYIRFLQ-----

Potri.005G230800.1/319-357 ALQQLVAP-F-------GK--TDTASVLMEAIGYIKFLQ-----

Potri.002G032400.1/319-358 ALQQLVAP-F-------GK--TDTASVLMEAIGYIKFLQ-----

Potri.007G020200.1/288-325 ALQRLVAP-Y-------GK--TDTASVLTEAIGYIQFL------

Potri.013G126800.1/33-72 ALQQLVSP-Y-------GK--TDTASVLMEAMEYIRFL------

Potri.019G112000.1/33-72 ALQQLVSP-Y-------GK--TDTASVLMEAMEYIRFL------

Potri.001G299300.1/160-197 ALQELVSP-F-------GK--TDTASVLHEAMGYIRFLQ-----

Potri.009G094300.1/160-197 SLQQLVSP-F-------GK--TDTASVLHEAMGYIRFLQ-----

Potri.010G098900.1/342-361 -----------------GR--TDTASVLYEAIQYIKYLQ-----

Potri.005G158100.1/314-353 ILQNLIPG-G------KG---KDAIVVLEEAIQYLKSL------

Potri.017G041000.1/221-257 CLRKVMPWIT-------TK--MDTATVLEEACRYIMNLQ-----

Potri.011G080000.1/189-225 TLRGIVPGGD--------Q--MNTVTVLDEAVRYLKSL------

Potri.006G037100.1/70-116 KLRGLLPC-N-------SK--TDKASLLAKVVQRVREL------

Potri.016G035400.1/71-117 KLRGLLLC-N-------SK--TDKASLLAKVVQRVREL------

Potri.002G105300.1/25-72 TLRTLLPN-P-------TK--TDKASLLAQVVHHVRDL------

Potri.010G130000.1/165-211 KLRSLLPS-T-------TK--TDKASLLAEVIQHVKEL------

Potri.008G116000.1/163-209 KLRSLLPS-T-------TK--TDKASLLAEVIQHVKEL------

Potri.004G055700.1/75-117 TLRGLVPC-T-------EK--MDKATLLAAVISQ----------

Potri.011G065500.1/39-80 TLRGLVPC-T-------EK--MDKATLLAAVIS-----------

Potri.013G117600.1/74-120 TLRSLVPG-T-------SK--MDKASLLAEVISHLKEL------

Potri.019G089000.1/74-120 TLRSLVPG-T-------RK--MDKASLLAEVIAHLKEL------

Potri.019G089300.1/47-70 TLKNRVPG------------------------------------

Potri.008G070800.1/69-115 ILRKLIPK-S-------EK--MDKAALLGSAIDHVKDL------

Potri.010G186700.1/69-115 ILRKLVPK-S-------EK--MDKAALLGSAIDHVKDL------

Potri.007G010500.1/61-107 TLRTILPN-L-------IK--VNKASVLEETIRCVKEL------

Potri.T155900.1/132-175 ALRSLVPN-I-------TK--MDKASIIGDAVLYVQELQ-----

Potri.009G005600.1/132-175 ALRSLVPN-I-------TK--MDKASIIGDAVLFVQELQ-----

Potri.008G161800.1/159-207 ALHSLLPH-D-------TK--KDKNSIMLMAAKKIQELE-----

Potri.010G077000.1/74-123 ALHSLLPL-G-------TK--KDKNSIMQMAAKRIQELE-----

Potri.004G029800.1/41-85 TLRALVPI-I-------TN--MNKGTIIEDAITYIQEL------

Potri.018G141500.1/54-99 ELRQAVPKIS--------K--LDKASTIKDAIDYIQDLQ-----

Potri.006G074600.1/57-99 ELRGAVPKIS--------K--LDKASTLKDAIVYIQDLQ-----

Potri.018G141600.1/55-99 ALREAVPRIS--------K--LDKASIIKDAIDYIQDLQ-----

Potri.006G074800.1/52-100 ALREAVPKIS--------K--LDKASIIKDAIGYIQDLQ-----

Potri.006G074700.1/59-102 ALREAVPKIS--------K--MDKASIIKDAIDYIQDLQ-----

Potri.018G141700.1/57-100 ALREAVPKIS--------K--LDKASVIKDAIKYIQDLQ-----

Potri.006G074900.1/57-99 ALRAVVPNIS--------K--MDKASIIKDAIDYIQEL------

Potri.018G141800.1/56-99 ALRAVVPNIS--------K--MDKASIIKDAIDYIQEL------

Potri.008G189600.1/234-276 ALRALVPKIS--------K--MDKAAILGDAIDYVGEL------

Potri.010G041500.1/115-157 SLSARVPKIS--------K--MDKAAILGDAIDYISEL------

Potri.015G105200.1/374-416 MLRSVVPKIS--------K--MDRASILGDAIEYLKEL------

Potri.012G106000.1/362-404 MLRSVVPKIS--------K--MDRASILGDAIDYLKEL------

Potri.002G108400.1/181-224 MLRSIVPKIS--------K--MDRTSILGDTIDYMKEL------

Potri.009G136300.1/336-379 ALRSLVPNIS--------K--LDRASILGDAIEFVKELQ-----

Potri.012G031800.1/89-140 VLRSLMPC-F-----YVKR--GDQASIIGGVVDYINELQ-----

Potri.015G022300.1/96-147 VLRSLMPC-F-----YVKR--GDQASIIGGVVDYINELQ-----

Potri.008G202900.1/2-49 VLRSLTPC-F-----YIKR--GDQASIIGGAIEFIKEL------

Potri.013G025900.1/140-191 VLRSLMPE-S-----YVQR--GDQASIIGGAINFVKELE-----

Potri.005G039800.1/142-193 VLRALMPE-S-----YVQR--GDQASIIGGAINFVKELE-----

Potri.002G101900.1/128-179 VLRSLMPP-S-----YVQR--GDQASIIGGAINFVKELE-----

Potri.005G071100.1/101-152 VIRSMMPP-S-----YVQR--PDQASIIGGAINFVKELE-----

Potri.007G097600.1/89-140 VIRSMLPP-S-----YVQR--ADQASIVGGAINFVKELE-----

Potri.003G093200.1/99-149 VLRSLMPE-S-----YVRR--GDQASIVGGAIEFVKEM------

Potri.001G141100.1/99-150 VLRSLMPE-S-----YVQR--GDQASIVGGAIEFVKELE-----

Potri.001G314400.1/210-261 VLRSLMPG-S-----YVQR--GDQASIIGGAIEFVRELE-----

Potri.017G054500.1/223-274 VLRSFMPG-S-----YVQR--GDQASIIGGAIEFVRELE-----

Potri.002G180300.1/200-251 SLRSFMPP-S-----YVQR--GDQASIIGGAIDFVKELE-----

Potri.014G106300.1/198-249 SLRSLMTP-S-----YIQK--GDQASIIGGAIDFVKELE-----

Potri.016G068500.1/260-306 ALRNLVPN-P-------SK--NDRASVVGEAIDYIKEL------

Potri.006G202100.1/259-305 ALRNLVPN-P-------SK--NDRASVVGDAINYIKEL------

Potri.009G023800.1/42-86 TLRDLIKN-P------STK--EDRATVIRDAIKYIIQL------

Potri.006G037600.1/70-122 SLRSLLPASD-----GMKK--LSIPSTISRVLKYIPELQ-----

Potri.016G037300.1/71-123 SLRSLLPA-A----DQRKK--LSIPYTVSRVLVYIPKLQ-----

Potri.006G037200.1/72-123 SLRSLLPA-A----DQMK---LSVPATVSRALKYLPELQ-----

Potri.015G134300.1/72-124 SLRALLPL-EF----IKGK--RSISDHMNEAVNYIKYLQ-----

Potri.012G132000.1/72-124 SLRALLPL-EF----IKGK--RSISDHMNESVNYIKYLQ-----

Potri.001G113400.1/80-129 SLRCLLPLKY-----LKGK--RSTSDHIHQTVYYIKHQ------

Potri.015G134400.1/75-127 SLRNLLPPDS-----IKGR--RSISDHMSEAVKCIKHLK-----

Potri.012G132100.1/153-204 SLRNLLPLES-----IKGK--RSISDHMNEAAKYIKHL------

Potri.011G053400.1/9-61 QLNSLVPHQS-----SREPV-LSLPDQLDEAASYIKRLQ-----

Potri.001G063000.1/9-61 QLNSLVPHQS-----SREPV-LSLPDQLDEAASYIKRLQ-----

Potri.004G044400.1/9-61 QLNSLVPHQS-----SREPV-LSVPDQLDEAASYIKRLQ-----

Potri.019G099300.1/24-74 SLNSLLPNQN-----FKEA--QPLPDQIDRAINYIKSLE-----

Potri.019G099400.1/31-81 SLNSLLPNQN-----FKEA--QPLPDQIDRAINYIKSLE-----

Potri.019G099500.1/40-90 SLNSLLPNQN-----FKEA--QPLPDQIDRAINYIKSLE-----

Potri.013G129800.1/22-72 KLNSLLPNQN-----FKEP--QPLPDQIDEAISHIKSLE-----

Potri.019G034700.1/67-116 KLNSLLPDKE-----STEK--QPLPDQIDEAISYIKTLE-----

Potri.012G079100.1/13-64 KLASLLPPHLF--KPSKDM--LSQQDQLELAACYIKQLR-----

Potri.015G074500.1/14-63 KLASLVPH-HFF-KPSKDM--LSQQDQLELAACYIKQM------

Potri.012G069500.1/22-59 KLQQLLPEIR---NRRSDK--VSAAKILQETCNYIKSL------

Potri.015G063300.1/22-59 KLQQLLPE-TR--NRRSEK--VSAAKALQETCNYIKSL------

Potri.017G081300.1/18-57 KLRQLLPE-IS--QRRSDK--VSASKVLQETCNYIRNL------

Potri.004G128400.1/23-57 KLRQLLPEIR---QRRSDK--VSASKVLQETCNYIKNL------

Potri.001G305100.1/89-136 NLHALLPQ-L------PPK--ADKSTIVDEAMNYIKTLQ-----

Potri.014G025900.1/84-127 SLHALLPQ-L------PAK--ADKSSIVDEAVKYIKTLQ-----

Potri.003G128000.1/446-492 ILKSIVPSIS--------K--VDKVSILDDTIQYLQELE-----

Potri.001G103600.1/431-478 ILKSIVPSIS--------KV-VDKVSILDETIEYLQELE-----

Potri.002G159400.1/465-501 VLRSMVPSIS--------E--IDKESILSDTINYLKQLE-----

Potri.008G165700.1/15-62 DLSSILEP-G-----RQAK--TDKPAILDDAIRVLNQL------

Potri.010G072900.1/70-117 DLSSVLEP-G-----RPAK--TDKPAILDDAIRVLNQL------

Potri.001G416600.1/107-154 ELGSILEP-G-----RTPK--TDKAAILVDAVRMVTQL------

Potri.011G132400.1/92-139 ELGSILDP-G-----RTPK--TDKAAILVDAVRIVTQL------

Potri.011G031000.1/87-133 ELGALLDP-G-----RPPK--VDKSAILVDAARMVTQL------

Potri.004G031800.1/87-133 ELGALLDP-G-----RPPK--VDKSAMLVDAARMVTQL------

Potri.009G129600.1/40-87 ELGNTLDP-D------RPK--NDKATILADTVQLLKDL------

Potri.004G168100.1/44-91 ELGNTLDP-D------RPK--NDKATILADTIQLLKDL------

Potri.005G113400.1/62-110 ELGTTLDP-D------RPK--NDKATILTDTIQVLKDL------

Potri.003G074400.1/31-80 ELASALEL-S------QPN--NGKASMLCETTRLLKDL------

Potri.015G142700.1/31-80 DLASALEL-S------EPN--TGKASILCETTRLLKDL------

| Potri.006G148800.1/221-265 | ALRSILPP-E-------AK--KDKASILTRTREYLTSL------ |
| --- | --- |
