# Supplementary material for: Characterization of the basic helix–loop–helix gene family and its tissue-differential expression in response to salt stress in poplar
Source: PeerJ. 2018 Mar 14;6:e4502. doi: 10.7717/peerj.4502 (PMC5857177; doi:10.7717/peerj.4502)
Supplement: Supplemental Information 13 [file peerj-06-4502-s013.doc]

**Annotations of bHLH protein sequence motifs**

| Name | Sequence | Description of InterProScan | Description of Pfam | Distribution | |
| --- | --- | --- | --- | --- | --- |
| motif1 | DYIHVRARRGQATDSHSLAERVRRERINERMKILQDLVPGC | Myc-type, basic helix-loop-helix (bHLH) domain (IPR011598) | Helix-loop-helix DNA-binding domain | | Group A, B, C, E, G, H, I, L. |
| motif2 | KHSVAERQRREKLNDRFYALRALVPNISKMDKASVLGDAIDYIKELQEQVKEL | Myc-type, basic helix-loop-helix (bHLH) domain (IPR011598) E-box/N-box specificity site | Helix-loop-helix DNA-binding domain | | Group D, F, H, J, K, L, M, N, P, Q, V, W, X, Y. |
| motif3 | KKTDKASMLDEIINYVKSLQRQVEFLSMK | Myc-type, basic helix-loop-helix (bHLH) domain (IPR011598) | No | | Group A, B, C, E, G, H, I, R, W. |
| motif4 | RRKRRRPRSIKNKEEVESQRMTHIAVERNRRKQMNEYLSVLRSLMPPSYVQRGDQASIIGGAINFVKELEQLLQSLEAQK | Myc-type, basic helix-loop-helix (bHLH) domain (IPR011598) E-box/N-box specificity site | Helix-loop-helix DNA-binding domain | | Group O. |
| motif5 | KICCERRPGQLSKJIEALESLGLTVLHANITTVGGRVLYTF | ACT domain (IPR002912) | No | | Group G, J, K, L, M, N, O, P, Q, R, T, V. |
| motif6 | KRDKADILDEAINYIKQLQEKVZKLKEKK | Myc-type, basic helix-loop-helix (bHLH) domain (IPR011598) | Helix-loop-helix DNA-binding domain | | Group A, C, G, I, J, K, L, O, Q, R, S, T, U, V. |
| motif7 | KKTSHKEIERNRREKINELYSSLRSLLPP | Myc-type, basic helix-loop-helix (bHLH) domain (IPR011598) | Helix-loop-helix DNA-binding domain | | Group G, H, I, K, P, Q, R, S, T, U, V. |
| motif8 | PQJKIHEIGSALEIVLTSGLDNQFLFYEIIRILHEEGVEVVSANFQVLGDSFFHIIHAQMKDSADG | Achaete-scute transcription factor-related (IPR015660) | No | | Group C, T. |
| motif9 | RSLLAKSAGIQTVVCIPTLSGVVELGSTELIPEDWGLVQHAKSLFGADS | Transcription factor MYC/MYB N-terminal (IPR025610) | bHLH-MYC and R2R3-MYB transcription factors N-terminal | | Group A, H, M, Q. |
| motif10 | DVEGAKQDLRSRGLCLVPISCTFAIASDNG | No | No | | Group B, X, Y. |
| motif11 | TEWFYLVSMTFSFSPGDGJPG | No | No | | Group A, N, Q. |
| motif12 | LQLAIAVRSEQWSYAIFWQAS | No | No | | Group M, Q. |
| motif13 | GQGVLGWGDGYYRGPKKTRKT | No | No | | Group B, Q. |
| motif14 | LLEEIRELKAEKNELREEKASLKADKEKLEQQLKALARPPS | No | No | | Group A, I. |
| motif15 | IAEVEVKIVGSDALJ | No | No | | Group A, B, I, K, M, N, O, P, Q. |
