# Supplementary material for: Characterization of the basic helix–loop–helix gene family and its tissue-differential expression in response to salt stress in poplar
Source: PeerJ. 2018 Mar 14;6:e4502. doi: 10.7717/peerj.4502 (PMC5857177; doi:10.7717/peerj.4502)
Supplement: Supplemental Information 14 [file peerj-06-4502-s014.doc]

Subcellular localization and gene coordinate of bHLH proteins

| Name | Gene ID | Subcellular localization | Chromosome | Transcript Start |
| --- | --- | --- | --- | --- |
| PtrbHLH1 | Potri.001G062900.1 | nucl | scaffold_1 | 4901700 |
| PtrbHLH2 | Potri.001G063000.1 | nucl | scaffold_1 | 4937901 |
| PtrbHLH3 | Potri.001G083500.1 | nucl | scaffold_1 | 6590377 |
| PtrbHLH4 | Potri.001G103600.1 | nucl | scaffold_1 | 8250635 |
| PtrbHLH5 | Potri.001G113400.1 | chlo | scaffold_1 | 9065467 |
| PtrbHLH6 | Potri.001G141100.1 | nucl | scaffold_1 | 11342732 |
| PtrbHLH7 | Potri.001G142200.1 | nucl | scaffold_1 | 11468524 |
| PtrbHLH8 | Potri.001G185900.1 | nucl | scaffold_1 | 16309624 |
| PtrbHLH9 | Potri.001G191800.1 | nucl | scaffold_1 | 17384029 |
| PtrbHLH10 | Potri.001G270000.1 | nucl | scaffold_1 | 27740118 |
| PtrbHLH11 | Potri.001G287200.1 | nucl | scaffold_1 | 29325151 |
| PtrbHLH12 | Potri.001G294300.1 | nucl | scaffold_1 | 29925642 |
| PtrbHLH13 | Potri.001G299300.1 | nucl | scaffold_1 | 30365962 |
| PtrbHLH14 | Potri.001G305100.1 | nucl | scaffold_1 | 30801398 |
| PtrbHLH15 | Potri.001G314400.1 | nucl | scaffold_1 | 32020286 |
| PtrbHLH16 | Potri.001G410600.1 | nucl | scaffold_1 | 43482832 |
| PtrbHLH17 | Potri.001G416600.1 | nucl | scaffold_1 | 44140016 |
| PtrbHLH18 | Potri.001G461000.1 | nucl | scaffold_1 | 49482946 |
| PtrbHLH19 | Potri.002G032400.1 | nucl | scaffold_2 | 2088970 |
| PtrbHLH20 | Potri.002G042000.1 | nucl | scaffold_2 | 2694371 |
| PtrbHLH21 | Potri.002G045400.1 | nucl | scaffold_2 | 2879435 |
| PtrbHLH22 | Potri.002G054100.1 | nucl | scaffold_2 | 3592072 |
| PtrbHLH23 | Potri.002G055400.1 | nucl | scaffold_2 | 3705303 |
| PtrbHLH24 | Potri.002G100100.1 | chlo | scaffold_2 | 7225353 |
| PtrbHLH25 | Potri.002G101900.1 | nucl | scaffold_2 | 7355732 |
| PtrbHLH26 | Potri.002G105300.1 | nucl | scaffold_2 | 7654242 |
| PtrbHLH27 | Potri.002G108400.1 | nucl | scaffold_2 | 7976030 |
| PtrbHLH28 | Potri.002G114700.1 | nucl | scaffold_2 | 8622936 |
| PtrbHLH29 | Potri.002G119200.1 | nucl | scaffold_2 | 8943221 |
| PtrbHLH30 | Potri.002G124400.1 | nucl | scaffold_2 | 9353395 |
| PtrbHLH31 | Potri.002G125000.1 | nucl | scaffold_2 | 9407564 |
| PtrbHLH32 | Potri.002G143300.1 | nucl | scaffold_2 | 10651063 |
| PtrbHLH33 | Potri.002G159400.1 | chlo | scaffold_2 | 11979027 |
| PtrbHLH34 | Potri.002G172100.1 | nucl | scaffold_2 | 13065986 |
| PtrbHLH35 | Potri.002G176900.1 | nucl | scaffold_2 | 13643953 |
| PtrbHLH36 | Potri.002G180300.1 | nucl | scaffold_2 | 14004400 |
| PtrbHLH37 | Potri.002G231700.1 | nucl | scaffold_2 | 22409991 |
| PtrbHLH38 | Potri.002G235400.1 | nucl | scaffold_2 | 22898104 |
| PtrbHLH39 | Potri.002G248500.1 | nucl | scaffold_2 | 23903060 |
| PtrbHLH40 | Potri.002G252800.1 | nucl | scaffold_2 | 24187022 |
| PtrbHLH41 | Potri.003G051600.1 | nucl | scaffold_3 | 7644752 |
| PtrbHLH42 | Potri.003G074400.1 | nucl | scaffold_3 | 10305460 |
| PtrbHLH43 | Potri.003G092200.1 | nucl | scaffold_3 | 11924022 |
| PtrbHLH44 | Potri.003G093200.1 | nucl | scaffold_3 | 12041835 |
| PtrbHLH45 | Potri.003G128000.1 | nucl | scaffold_3 | 14830998 |
| PtrbHLH46 | Potri.003G147300.1 | cyto | scaffold_3 | 16267723 |
| PtrbHLH47 | Potri.003G164500.1 | nucl | scaffold_3 | 17512828 |
| PtrbHLH48 | Potri.003G207200.1 | nucl | scaffold_3 | 20790086 |
| PtrbHLH49 | Potri.004G029100.1 | nucl | scaffold_4 | 2118907 |
| PtrbHLH50 | Potri.004G029800.1 | nucl | scaffold_4 | 2164085 |
| PtrbHLH51 | Potri.004G031800.1 | nucl | scaffold_4 | 2299675 |
| PtrbHLH52 | Potri.004G044400.1 | nucl | scaffold_4 | 3372150 |
| PtrbHLH53 | Potri.004G055700.1 | nucl | scaffold_4 | 4406014 |
| PtrbHLH54 | Potri.004G088900.1 | nucl | scaffold_4 | 7529439 |
| PtrbHLH55 | Potri.004G099400.1 | nucl | scaffold_4 | 8599017 |
| PtrbHLH56 | Potri.004G112900.1 | nucl | scaffold_4 | 10227500 |
| PtrbHLH57 | Potri.004G128400.1 | nucl | scaffold_4 | 13628912 |
| PtrbHLH58 | Potri.004G156000.1 | nucl | scaffold_4 | 17799366 |
| PtrbHLH59 | Potri.004G168100.1 | nucl | scaffold_4 | 18743947 |
| PtrbHLH60 | Potri.005G001800.1 | nucl | scaffold_5 | 107969 |
| PtrbHLH61 | Potri.005G039800.1 | nucl | scaffold_5 | 2892681 |
| PtrbHLH62 | Potri.005G053500.1 | nucl | scaffold_5 | 3814251 |
| PtrbHLH63 | Potri.005G060900.1 | nucl | scaffold_5 | 4268801 |
| PtrbHLH64 | Potri.005G071100.1 | nucl | scaffold_5 | 5128167 |
| PtrbHLH65 | Potri.005G095400.1 | nucl | scaffold_5 | 7140559 |
| PtrbHLH66 | Potri.005G113400.1 | nucl | scaffold_5 | 8722917 |
| PtrbHLH67 | Potri.005G121900.1 | nucl | scaffold_5 | 9482402 |
| PtrbHLH68 | Potri.005G138900.1 | nucl | scaffold_5 | 11546854 |
| PtrbHLH69 | Potri.005G139700.1 | nucl | scaffold_5 | 11648940 |
| PtrbHLH70 | Potri.005G146500.1 | nucl | scaffold_5 | 12875562 |
| PtrbHLH71 | Potri.005G158100.1 | nucl | scaffold_5 | 15860686 |
| PtrbHLH72 | Potri.005G207200.1 | nucl | scaffold_5 | 22153104 |
| PtrbHLH73 | Potri.005G208600.1 | nucl | scaffold_5 | 22260946 |
| PtrbHLH74 | Potri.005G217800.1 | nucl | scaffold_5 | 23030899 |
| PtrbHLH75 | Potri.005G221100.1 | nucl | scaffold_5 | 23273962 |
| PtrbHLH76 | Potri.005G230800.1 | nucl | scaffold_5 | 23942678 |
| PtrbHLH77 | Potri.006G037100.1 | nucl | scaffold_6 | 2552810 |
| PtrbHLH78 | Potri.006G037200.1 | nucl | scaffold_6 | 2563601 |
| PtrbHLH79 | Potri.006G037600.1 | nucl | scaffold_6 | 2611375 |
| PtrbHLH80 | Potri.006G057200.1 | nucl | scaffold_6 | 4158885 |
| PtrbHLH81 | Potri.006G057600.1 | nucl | scaffold_6 | 4204832 |
| PtrbHLH82 | Potri.006G074600.1 | nucl | scaffold_6 | 5563549 |
| PtrbHLH83 | Potri.006G074700.1 | nucl | scaffold_6 | 5587755 |
| PtrbHLH84 | Potri.006G074800.1 | nucl | scaffold_6 | 5595383 |
| PtrbHLH85 | Potri.006G074900.1 | nucl | scaffold_6 | 5618411 |
| PtrbHLH86 | Potri.006G102600.1 | nucl | scaffold_6 | 7938552 |
| PtrbHLH87 | Potri.006G135600.1 | golg | scaffold_6 | 11199861 |
| PtrbHLH88 | Potri.006G148800.1 | nucl | scaffold_6 | 12897663 |
| PtrbHLH89 | Potri.006G186600.1 | nucl | scaffold_6 | 20100199 |
| PtrbHLH90 | Potri.006G202100.1 | nucl | scaffold_6 | 21700815 |
| PtrbHLH91 | Potri.007G009400.1 | nucl | scaffold_7 | 738377 |
| PtrbHLH92 | Potri.007G010500.1 | chlo | scaffold_7 | 810863 |
| PtrbHLH93 | Potri.007G020200.1 | nucl | scaffold_7 | 1516807 |
| PtrbHLH94 | Potri.007G023600.1 | nucl | scaffold_7 | 1772491 |
| PtrbHLH95 | Potri.007G044600.1 | nucl | scaffold_7 | 3917573 |
| PtrbHLH96 | Potri.007G097600.1 | nucl | scaffold_7 | 12375888 |
| PtrbHLH97 | Potri.007G108000.1 | nucl | scaffold_7 | 13167982 |
| PtrbHLH98 | Potri.008G052000.1 | chlo | scaffold_8 | 3072962 |
| PtrbHLH99 | Potri.008G070800.1 | nucl | scaffold_8 | 4368197 |
| PtrbHLH100 | Potri.008G112000.1 | nucl | scaffold_8 | 7141505 |
| PtrbHLH101 | Potri.008G113200.1 | nucl | scaffold_8 | 7225402 |
| PtrbHLH102 | Potri.008G116000.1 | nucl | scaffold_8 | 7440426 |
| PtrbHLH103 | Potri.008G161800.1 | nucl | scaffold_8 | 11004417 |
| PtrbHLH104 | Potri.008G165700.1 | chlo | scaffold_8 | 11294782 |
| PtrbHLH105 | Potri.008G189600.1 | nucl | scaffold_8 | 13141115 |
| PtrbHLH106 | Potri.008G190800.1 | nucl | scaffold_8 | 13246812 |
| PtrbHLH107 | Potri.008G202900.1 | nucl | scaffold_8 | 14522687 |
| PtrbHLH108 | Potri.009G005600.1 | nucl | scaffold_9 | 1046640 |
| PtrbHLH109 | Potri.009G023800.1 | nucl | scaffold_9 | 3534904 |
| PtrbHLH110 | Potri.009G064700.1 | nucl | scaffold_9 | 6545643 |
| PtrbHLH111 | Potri.009G081400.1 | nucl | scaffold_9 | 7740867 |
| PtrbHLH112 | Potri.009G089000.1 | nucl | scaffold_9 | 8207117 |
| PtrbHLH113 | Potri.009G094300.1 | nucl | scaffold_9 | 8537506 |
| PtrbHLH114 | Potri.009G117300.1 | nucl | scaffold_9 | 9904054 |
| PtrbHLH115 | Potri.009G129600.1 | nucl | scaffold_9 | 10576966 |
| PtrbHLH116 | Potri.009G136300.1 | nucl | scaffold_9 | 10960459 |
| PtrbHLH117 | Potri.010G040000.1 | nucl | scaffold_10 | 6730377 |
| PtrbHLH118 | Potri.010G041500.1 | nucl | scaffold_10 | 7147976 |
| PtrbHLH119 | Potri.010G072900.1 | nucl | scaffold_10 | 9958995 |
| PtrbHLH120 | Potri.010G077000.1 | cyto | scaffold_10 | 10275116 |
| PtrbHLH121 | Potri.010G098900.1 | nucl | scaffold_10 | 12053774 |
| PtrbHLH122 | Potri.010G130000.1 | nucl | scaffold_10 | 14508429 |
| PtrbHLH123 | Potri.010G136100.1 | nucl | scaffold_10 | 14970791 |
| PtrbHLH124 | Potri.010G137600.1 | nucl | scaffold_10 | 15061893 |
| PtrbHLH125 | Potri.010G186700.1 | nucl | scaffold_10 | 18298179 |
| PtrbHLH126 | Potri.010G208600.1 | nucl | scaffold_10 | 19822713 |
| PtrbHLH127 | Potri.011G031000.1 | nucl | scaffold_11 | 2587364 |
| PtrbHLH128 | Potri.011G033000.1 | nucl | scaffold_11 | 2706931 |
| PtrbHLH129 | Potri.011G053400.1 | cyto_nucl | scaffold_11 | 4621028 |
| PtrbHLH130 | Potri.011G065500.1 | nucl | scaffold_11 | 6002010 |
| PtrbHLH131 | Potri.011G080000.1 | nucl | scaffold_11 | 7934591 |
| PtrbHLH132 | Potri.011G129500.1 | nucl | scaffold_11 | 15453992 |
| PtrbHLH133 | Potri.011G132400.1 | nucl | scaffold_11 | 15728883 |
| PtrbHLH134 | Potri.011G157700.1 | nucl | scaffold_11 | 17507817 |
| PtrbHLH135 | Potri.012G031800.1 | nucl | scaffold_12 | 2825815 |
| PtrbHLH136 | Potri.012G055700.1 | nucl | scaffold_12 | 5729096 |
| PtrbHLH137 | Potri.012G065000.1 | nucl | scaffold_12 | 8379760 |
| PtrbHLH138 | Potri.012G069500.1 | nucl | scaffold_12 | 9259907 |
| PtrbHLH139 | Potri.012G072700.1 | nucl | scaffold_12 | 9823077 |
| PtrbHLH140 | Potri.012G079100.1 | nucl | scaffold_12 | 10500236 |
| PtrbHLH141 | Potri.012G104900.1 | nucl | scaffold_12 | 12894761 |
| PtrbHLH142 | Potri.012G106000.1 | nucl | scaffold_12 | 12987418 |
| PtrbHLH143 | Potri.012G132000.1 | nucl | scaffold_12 | 14836487 |
| PtrbHLH144 | Potri.012G132100.1 | chlo | scaffold_12 | 14850888 |
| PtrbHLH145 | Potri.013G001300.1 | nucl | scaffold_13 | 86122 |
| PtrbHLH146 | Potri.013G025900.1 | nucl | scaffold_13 | 1714147 |
| PtrbHLH147 | Potri.013G041000.1 | cyto | scaffold_13 | 2870421 |
| PtrbHLH148 | Potri.013G107500.1 | nucl | scaffold_13 | 12107520 |
| PtrbHLH149 | Potri.013G117600.1 | nucl | scaffold_13 | 13089521 |
| PtrbHLH150 | Potri.013G126800.1 | nucl | scaffold_13 | 13930841 |
| PtrbHLH151 | Potri.013G129800.1 | nucl | scaffold_13 | 14156936 |
| PtrbHLH152 | Potri.014G017100.1 | nucl | scaffold_14 | 1644259 |
| PtrbHLH153 | Potri.014G025800.1 | nucl | scaffold_14 | 2209016 |
| PtrbHLH154 | Potri.014G025900.1 | nucl | scaffold_14 | 2214843 |
| PtrbHLH155 | Potri.014G027300.1 | nucl | scaffold_14 | 2315639 |
| PtrbHLH156 | Potri.014G066500.1 | nucl | scaffold_14 | 5402159 |
| PtrbHLH157 | Potri.014G099700.1 | nucl | scaffold_14 | 7767124 |
| PtrbHLH158 | Potri.014G103700.1 | nucl | scaffold_14 | 8174722 |
| PtrbHLH159 | Potri.014G106300.1 | nucl | scaffold_14 | 8368556 |
| PtrbHLH160 | Potri.014G111400.1 | nucl | scaffold_14 | 8714454 |
| PtrbHLH161 | Potri.014G148900.1 | nucl | scaffold_14 | 11386746 |
| PtrbHLH162 | Potri.014G150600.1 | nucl | scaffold_14 | 11543574 |
| PtrbHLH163 | Potri.015G022300.1 | nucl | scaffold_15 | 1721394 |
| PtrbHLH164 | Potri.015G046300.1 | nucl | scaffold_15 | 4669993 |
| PtrbHLH165 | Potri.015G048000.1 | nucl | scaffold_15 | 4970293 |
| PtrbHLH166 | Potri.015G063300.1 | nucl | scaffold_15 | 8702197 |
| PtrbHLH167 | Potri.015G068100.1 | nucl | scaffold_15 | 9265479 |
| PtrbHLH168 | Potri.015G074500.1 | cyto | scaffold_15 | 9917247 |
| PtrbHLH169 | Potri.015G104200.1 | nucl | scaffold_15 | 12228818 |
| PtrbHLH170 | Potri.015G105200.1 | nucl | scaffold_15 | 12317939 |
| PtrbHLH171 | Potri.015G134300.1 | nucl | scaffold_15 | 14396987 |
| PtrbHLH172 | Potri.015G134400.1 | nucl | scaffold_15 | 14408685 |
| PtrbHLH173 | Potri.015G142700.1 | cyto | scaffold_15 | 14907870 |
| PtrbHLH174 | Potri.016G035400.1 | nucl | scaffold_16 | 2066676 |
| PtrbHLH175 | Potri.016G037300.1 | nucl | scaffold_16 | 2278010 |
| PtrbHLH176 | Potri.016G050500.1 | nucl | scaffold_16 | 3216957 |
| PtrbHLH177 | Potri.016G051100.1 | nucl | scaffold_16 | 3264767 |
| PtrbHLH178 | Potri.016G068500.1 | nucl | scaffold_16 | 4899203 |
| PtrbHLH179 | Potri.016G120800.1 | nucl | scaffold_16 | 12555408 |
| PtrbHLH180 | Potri.017G041000.1 | nucl | scaffold_17 | 3348713 |
| PtrbHLH181 | Potri.017G054500.1 | nucl | scaffold_17 | 4816204 |
| PtrbHLH182 | Potri.017G081300.1 | chlo | scaffold_17 | 9548795 |
| PtrbHLH183 | Potri.017G101700.1 | nucl | scaffold_17 | 11871090 |
| PtrbHLH184 | Potri.017G115300.1 | nucl | scaffold_17 | 13058828 |
| PtrbHLH185 | Potri.017G126800.1 | nucl | scaffold_17 | 13869380 |
| PtrbHLH186 | Potri.018G083700.1 | chlo | scaffold_18 | 11057827 |
| PtrbHLH187 | Potri.018G109500.1 | nucl | scaffold_18 | 13582853 |
| PtrbHLH188 | Potri.018G141500.1 | nucl | scaffold_18 | 16120443 |
| PtrbHLH189 | Potri.018G141600.1 | nucl | scaffold_18 | 16124602 |
| PtrbHLH190 | Potri.018G141700.1 | nucl | scaffold_18 | 16143678 |
| PtrbHLH191 | Potri.018G141800.1 | nucl | scaffold_18 | 16183690 |
| PtrbHLH192 | Potri.019G034700.1 | chlo | scaffold_19 | 3945668 |
| PtrbHLH193 | Potri.019G079900.1 | nucl | scaffold_19 | 11379293 |
| PtrbHLH194 | Potri.019G089000.1 | nucl | scaffold_19 | 12075920 |
| PtrbHLH195 | Potri.019G089300.1 | cyto | scaffold_19 | 12101589 |
| PtrbHLH196 | Potri.019G099300.1 | mito | scaffold_19 | 12917850 |
| PtrbHLH197 | Potri.019G099400.1 | mito | scaffold_19 | 12925550 |
| PtrbHLH198 | Potri.019G099500.1 | mito | scaffold_19 | 12932112 |
| PtrbHLH199 | Potri.019G112000.1 | nucl | scaffold_19 | 14105847 |
| PtrbHLH200 | Potri.T107900.1 | nucl | scaffold_162 | 69786 |
| PtrbHLH201 | Potri.T155900.1 | nucl | scaffold_645 | 2257 |
| PtrbHLH202 | Potri.T179100.1 | nucl | scaffold_2029 | 327 |
