# Supplementary material for: Characterization of the basic helix–loop–helix gene family and its tissue-differential expression in response to salt stress in poplar
Source: PeerJ. 2018 Mar 14;6:e4502. doi: 10.7717/peerj.4502 (PMC5857177; doi:10.7717/peerj.4502)
Supplement: Supplemental Information 15 — Sample: name of sequencing sample. Total reads: the number of reads after filtering low quality reads. Length: average length of reads. Total mapped: the number of reads mapped to the reference genome. Multiple mapped: the number of reads mapped to the reference genome with multiple positions. Uniquely mapped: the number of reads mapped to the reference genome with unique position. [file peerj-06-4502-s015.doc]

The results of sequencing

| Sample | Total reads | Length | | Total mapped | | Multiple mapped | Uniquely mapped |
| --- | --- | --- | --- | --- | --- | --- | --- |
| L(C)1 | 38315264 | | 146.67 | | 36416359 (95.044%) | 2926529 (7.63802%) | 33489830 (87.406%) |
| L(C)2 | 36595926 | | 146.58 | | 34399275 (93.9976%) | 3413644 (9.32793%) | 30985631 (84.6696%) |
| S(C)1 | 31781706 | | 146.47 | | 29955309 (94.2533%) | 1968014 (6.19229%) | 27987295 (88.061%) |
| S(C)2 | 33647394 | | 146.58 | | 31521944 (93.6832%) | 2195502 (6.52503%) | 29326442 (87.1581%) |
| R(C)1 | 34625636 | | 146.44 | | 32136097 (92.8101%) | 2125278 (6.13787%) | 30010819 (86.6723%) |
| R(C)2 | 33749618 | | 146.43 | | 31190683 (92.4179%) | 2024681 (5.99912%) | 29166002 (86.4188%) |
| L(T)1 | 37887356 | | 146.83 | | 36071625 (95.2076%) | 3016848 (7.96268%) | 33054777 (87.2449%) |
| L(T)2 | 35488840 | | 146.61 | | 33807126 (95.2613%) | 2747750 (7.74257%) | 31059376 (87.5187%) |
| S(T)1 | 37365514 | | 146.18 | | 34943474 (93.518%) | 2327136 (6.22803%) | 32616338 (87.2899%) |
| S(T)2 | 36027004 | | 146.45 | | 33837097 (93.9215%) | 2256208 (6.26255%) | 31580889 (87.6589%) |
| R(T)1 | 35274634 | | 146.19 | | 27269202 (77.3054%) | 2714515 (7.69537%) | 24554687 (69.61%) |
| R(T)2 | 34898004 | | 146.31 | | 28199099 (80.8043%) | 2814432 (8.06474%) | 25384667 (72.7396%) |
