# Supplementary material for: Characterization of the basic helix–loop–helix gene family and its tissue-differential expression in response to salt stress in poplar
Source: PeerJ. 2018 Mar 14;6:e4502. doi: 10.7717/peerj.4502 (PMC5857177; doi:10.7717/peerj.4502)
Supplement: Supplemental Information 16 [file peerj-06-4502-s016.doc]

Tissue-differently expressed gene list without salt treatment

| Shared genes in root-leaf and root-stem pairs | Shared genes in leaf-root and leaf-stem pairs | Shared genes in stem-leaf and stem-root pairs | Shared genes in leaf-root leaf-stem and root-stem pairs |
| --- | --- | --- | --- |
| Potri.012G104900.1 | Potri.007G009400.1 | Potri.007G023600.1 | Potri.007G009400.1 |
| Potri.007G023600.1 | Potri.002G248500.1 | Potri.002G055400.1 | Potri.002G248500.1 |
| Potri.009G117300.1 | Potri.013G129800.1 | Potri.005G207200.1 | Potri.005G039800.1 |
| Potri.014G066500.1 | Potri.005G039800.1 | Potri.016G051100.1 | Potri.002G108400.1 |
| Potri.012G055700.1 | Potri.002G108400.1 | Potri.014G066500.1 | Potri.006G186600.1 |
| Potri.016G051100.1 | Potri.006G186600.1 | Potri.012G104900.1 | Potri.018G109500.1 |
| Potri.017G115300.1 | Potri.009G081400.1 | Potri.003G207200.1 | Potri.005G230800.1 |
| Potri.018G141700.1 | Potri.018G109500.1 | Potri.009G117300.1 | Potri.019G089000.1 |
| Potri.003G207200.1 | Potri.019G112000.1 | Potri.019G089000.1 | Potri.002G143300.1 |
| Potri.010G186700.1 | Potri.005G230800.1 | Potri.002G143300.1 | Potri.010G186700.1 |
| Potri.002G143300.1 | Potri.003G092200.1 | Potri.018G141800.1 | Potri.003G207200.1 |
| Potri.005G001800.1 | Potri.002G235400.1 | Potri.010G186700.1 | Potri.016G051100.1 |
| Potri.019G089000.1 | Potri.002G114700.1 | Potri.012G055700.1 | Potri.012G055700.1 |
| Potri.008G070800.1 | Potri.018G083700.1 | Potri.006G186600.1 | Potri.014G066500.1 |
| Potri.012G106000.1 | Potri.004G168100.1 | Potri.018G109500.1 | Potri.009G117300.1 |
| Potri.005G230800.1 | Potri.006G202100.1 | Potri.002G108400.1 | Potri.007G023600.1 |
| Potri.006G074900.1 | Potri.019G089000.1 | Potri.002G248500.1 | Potri.012G104900.1 |
| Potri.001G410600.1 | Potri.014G111400.1 | Potri.005G230800.1 | Potri.002G055400.1 |
| Potri.004G055700.1 | Potri.002G143300.1 | Potri.002G032400.1 | Potri.005G207200.1 |
| Potri.018G109500.1 | Potri.010G186700.1 | Potri.001G063000.1 |  |
| Potri.002G176900.1 | Potri.003G207200.1 | Potri.007G009400.1 |  |
| Potri.006G186600.1 | Potri.016G051100.1 | Potri.005G039800.1 |  |
| Potri.007G020200.1 | Potri.012G055700.1 |  |  |
| Potri.002G108400.1 | Potri.014G066500.1 |  |  |
| Potri.005G039800.1 | Potri.009G117300.1 |  |  |
| Potri.015G134300.1 | Potri.005G121900.1 |  |  |
| Potri.002G248500.1 | Potri.007G023600.1 |  |  |
| Potri.010G098900.1 | Potri.012G104900.1 |  |  |
| Potri.010G077000.1 | Potri.002G055400.1 |  |  |
| Potri.012G132100.1 | Potri.005G207200.1 |  |  |
| Potri.007G009400.1 | Potri.001G314400.1 |  |  |
| Potri.T155900.1 | Potri.003G093200.1 |  |  |
| Potri.012G132000.1 | Potri.017G054500.1 |  |  |
| Potri.007G097600.1 | Potri.005G071100.1 |  |  |
| Potri.005G095400.1 |  |  |  |
| Potri.017G126800.1 |  |  |  |
| Potri.001G113400.1 |  |  |  |
| Potri.002G055400.1 |  |  |  |
| Potri.005G207200.1 |  |  |  |
| Potri.015G104200.1 |  |  |  |
