# Supplementary material for: Characterization of the basic helix–loop–helix gene family and its tissue-differential expression in response to salt stress in poplar
Source: PeerJ. 2018 Mar 14;6:e4502. doi: 10.7717/peerj.4502 (PMC5857177; doi:10.7717/peerj.4502)
Supplement: Supplemental Information 18 [file peerj-06-4502-s018.doc]

Annotations of differentially expressed genes

|  | ID | Best-hit-arabi-name |
| --- | --- | --- |
| Gene differently expressed in leaf, root, and stem without salt treatment | Potri.007G009400.1 | AT4G37850.1 |
|  | Potri.002G248500.1 | AT3G07340.1 |
|  | Potri.005G039800.1 | AT1G72210.1 |
|  | Potri.002G108400.1 | AT5G65640.1 |
|  | Potri.006G186600.1 | AT2G24260.1 |
|  | Potri.018G109500.1 | AT2G24260.1 |
|  | Potri.005G230800.1 | AT1G27660.1 |
|  | Potri.019G089000.1 | AT1G68810.1 |
|  | Potri.002G143300.1 | AT4G00050.1 |
|  | Potri.010G186700.1 | AT2G40200.1 |
|  | Potri.003G207200.1 | AT1G51140.1 |
|  | Potri.016G051100.1 | AT3G57800.2 |
|  | Potri.012G055700.1 | AT1G73830.1 |
|  | Potri.014G066500.1 | AT4G00050.1 |
|  | Potri.009G117300.1 | AT4G34530.1 |
|  | Potri.007G023600.1 | AT2G18300.2 |
|  | Potri.012G104900.1 | AT5G50915.1 |
|  | Potri.002G055400.1 | AT2G43010.2 |
|  | Potri.005G207200.1 | AT2G43010.2 |
| Differential expression genes in response to salinity | Potri.005G121900.1 | AT2G18300.1 |
|  | Potri.017G081300.1 | AT3G28857.1 |
|  | Potri.007G023600.1 | AT2G18300.2 |
|  | Potri.009G117300.1 | AT4G34530.1 |
|  | Potri.014G103700.1 | AT1G32640.1 |
|  | Potri.003G093200.1 | AT5G46690.1 |
|  | Potri.012G104900.1 | AT5G50915.1 |
|  | Potri.017G115300.1 | AT1G25330.1 |
|  | Potri.005G207200.1 | AT2G43010.2 |
|  | Potri.008G070800.1 | AT2G40200.1 |
|  | Potri.016G051100.1 | AT3G57800.2 |
|  | Potri.001G142200.1 | AT1G32640.1 |
|  | Potri.004G168100.1 | AT3G19860.2 |
|  | Potri.014G111400.1 | AT1G09530.2 |
|  | Potri.005G208600.1 | AT4G09820.1 |
|  | Potri.010G186700.1 | AT2G40200.1 |
|  | Potri.018G109500.1 | AT2G24260.1 |
|  | Potri.014G106300.1 | AT2G46810.1 |
|  | Potri.002G054100.1 | AT4G09820.1 |
|  | Potri.009G081400.1 | AT4G37850.1 |
|  | Potri.002G248500.1 | AT3G07340.1 |
|  | Potri.015G074500.1 | AT4G20970.1 |
|  | Potri.001G141100.1 | AT5G46690.1 |
|  | Potri.012G055700.1 | AT1G73830.1 |
|  | Potri.013G025900.1 | AT1G72210.1 |
|  | Potri.004G156000.1 | AT4G34530.1 |
|  | Potri.010G098900.1 | AT1G31050.1 |
|  | Potri.007G097600.1 | AT1G72210.1 |
|  | Potri.008G116000.1 | AT1G68810.1 |
|  | Potri.007G009400.1 | AT4G37850.1 |
|  | Potri.002G159400.1 | AT5G41315.1 |
|  | Potri.002G143300.1 | AT4G00050.1 |
|  | Potri.005G230800.1 | AT1G27660.1 |
|  | Potri.002G108400.1 | AT5G65640.1 |
|  | Potri.005G071100.1 | AT1G72210.1 |
|  | Potri.002G114700.1 | AT1G10120.1 |
|  | Potri.010G130000.1 | AT1G68810.1 |
|  | Potri.007G020200.1 | AT1G27660.1 |
|  | Potri.010G136100.1 | AT1G68920.3 |
|  | Potri.018G083700.1 | AT4G29100.1 |
|  | Potri.010G137600.1 | AT1G69010.1 |
|  | Potri.011G031000.1 | AT5G54680.1 |
|  | Potri.011G080000.1 | AT1G29950.2 |
|  | Potri.005G221100.1 | AT4G16430.1 |
|  | Potri.012G065000.1 | AT5G08130.2 |
|  | Potri.002G172100.1 | AT1G01260.3 |
|  | Potri.018G141800.1 | AT5G57150.1 |
|  | Potri.019G089000.1 | AT1G68810.1 |
|  | Potri.008G161800.1 | AT5G43650.1 |
|  | Potri.001G410600.1 | AT3G20640.1 |
|  | Potri.002G176900.1 | AT1G32640.1 |
|  | Potri.009G064700.1 | AT2G42280.1 |
|  | Potri.006G202100.1 | AT2G31220.1 |
|  | Potri.008G112000.1 | AT1G69010.1 |
|  | Potri.010G077000.1 | AT5G43650.1 |
|  | Potri.008G190800.1 | AT1G59640.1 |
|  | Potri.004G088900.1 | AT5G37800.1 |
|  | Potri.015G142700.1 | AT3G47640.2 |
|  | Potri.016G068500.1 | AT2G31210.1 |
|  | Potri.001G287200.1 | AT4G37850.1 |
|  | Potri.018G141500.1 | AT5G57150.2 |
|  | Potri.005G095400.1 | AT4G37850.1 |
|  | Potri.006G148800.1 | AT5G56960.1 |
|  | Potri.013G129800.1 | AT4G20970.1 |
|  | Potri.019G099300.1 | AT4G20970.1 |
|  | Potri.017G041000.1 | AT3G21330.1 |
|  | Potri.019G099500.1 | AT4G20970.1 |
|  | Potri.003G207200.1 | AT1G51140.1 |
|  | Potri.014G066500.1 | AT4G00050.1 |
|  | Potri.005G039800.1 | AT1G72210.1 |
|  | Potri.010G072900.1 | AT4G14410.2 |
|  | Potri.005G158100.1 | AT5G50010.1 |
|  | Potri.006G186600.1 | AT2G24260.1 |
|  | Potri.008G165700.1 | AT4G14410.1 |
